# Clinical integration of germline findings from a tumor testing precision medicine program

**DOI:** 10.1186/s12885-025-13487-4

**Published:** 2025-01-30

**Authors:** Maria Carolina Sanabria-Salas, Nina C. Anggala, Brittany Gillies, Kirsten M. Farncombe, Renee Hofstedter, Larissa Peck, Helia Purnaghshband, Laura Redondo, Emily Thain, Wei Xu, Peter Sabatini, Philippe L. Bedard, Raymond H. Kim

**Affiliations:** 1 Department of Medicine, Division of Medical Oncology and Hematology, Princess Margaret Cancer Centre, University Health Network, University of Toronto, Toronto, ON Canada; 2 Mount Sinai Hospital, Sinai Health System, Toronto, ON Canada; 3 Bhalwani Familial Cancer Clinic, Princess Margaret Cancer Centre, University Health Network, Toronto, ON Canada; 4 Toronto General Hospital Research Institute, University Health Network, Toronto, ON Canada; 5 Ontario Institute for Cancer Research, Toronto, ON Canada; 6 Biostatistics Department, Dalla Lana School of Public Health, University Health Network, University of Toronto, Toronto, ON Canada; 7 Division of Genome Diagnostics, Department of Laboratory Medicine and Pathobiology, University Health Network, University of Toronto, Toronto, ON Canada; 8 Division of Medical Oncology and Hematology, Princess Margaret Cancer Centre, University Health Network, Sinai Health System, Toronto, ON Canada; 9 Division of Clinical and Metabolic Genetics, The Hospital for Sick Children, Toronto, ON Canada

**Keywords:** Precision medicine, Biomarkers, tumor, Genetic predisposition testing, Genetic counseling, High-throughput nucleotide sequencing, Molecular tumor board

## Abstract

**Background:**

Integrating germline genetic testing (GGT) recommendations from tumor testing into hereditary cancer clinics and precision oncology trials presents challenges that require multidisciplinary expertise and infrastructure. While there have been advancements in standardizing molecular tumor boards, the implementation of tumor profiling for germline-focused assessments has only recently gained momentum. However, this progress remains inconsistent across institutions, largely owing to a lack of systematic approaches for managing these findings. This study outlines the development of a clinical pathway for identifying potential germline variants from an institutional tumor-sequencing research program at Princess Margaret Cancer Centre.

**Methods:**

Between August 2022 and August 2023, a clinical pathway led by a germline Molecular Tumor Board (gMTB) was established to review tumor genetic variants (TGVs) flagged as potential germline findings in patients with advanced cancer via a multigene panel. Eligibility for hereditary cancer syndrome investigation (‘germline criteria’) followed Cancer Care Ontario’s Hereditary Cancer Testing Criteria and clinical judgment. Germline-focused analysis of TGVs followed the European Society of Medical Oncology guidelines and similar published criteria (‘tumor-only criteria’).

**Results:**

Of 243 tumor profiles, 83 (34.2%) had at least one TGV flagged by the genetic laboratory as potentially germline and were therefore referred to the gMTB for further review. Among these 83 cases, 47 (56.6%) met ‘germline criteria’ for GGT, regardless of the TGV assessment. A total of 127 TGVs were assessed in these 83 cases, of which 44 (34.6%) were considered *germline relevant*. Tier I TGVs, interpreted as pathogenic/likely pathogenic (P/LP) and found in most- or standard-actionable genes with high germline conversion rates (GCRs) in any context, were more likely to be considered *germline relevant* (*p*-value < 0.05). One confirmed germline variant was identified in nine patients meeting solely ‘tumor-only criteria’. Overall, 27/44 *germline relevant* TGVs underwent germline testing. We found a germline P/LP variant in 9 cases of the entire cohort, with a GCR of 33% (9/27).

**Conclusions:**

Incorporating genetic counselors into gMTBs enhanced the integration of research findings into clinical care and improved the detection of disease-causing variants in patients outside traditional testing criteria.

**Supplementary Information:**

The online version contains supplementary material available at 10.1186/s12885-025-13487-4.

## Background

Tumor genetic testing (TGT) has evolved from analyzing specific hotspot mutations [1–3] to using multigene sequencing panels for tumor genomic profiling [4, 5], aiming to identify therapeutic targets for patients with cancer, particularly advanced disease [6–8]. Such sequencing may uncover clinically actionable germline variants in moderate- and high-penetrance cancer susceptibility genes (CSGs), some with indications for FDA-approved therapies, clinical trials, and inherited cancer risk assessment for patients and their relatives [6, 9–15]. While numerous clinical trials are conducting TGT, the incorporation of these findings into clinical genetics care varies among centres.

TGT can be approached through paired tumor-normal and tumor-only testing. Paired tumor-normal testing sequences genetic data from matching samples to accurately identify somatic and germline variants by subtracting germline variants from tumor data. Tumor-only testing analyzes genetic information from the tumor sample alone (with mixed normal and tumor cells), making it challenging to distinguish somatic from germline origins [12, 15], thus limiting its clinical use for genetic cancer risk assessment [15].

The European Society of Medical Oncology (ESMO) Precision Medicine Working Group Germline Subgroup has generated recommendations for germline-focused analysis after a tumor-only sequencing approach for actionable genes based on the likelihood of germline origin [4, 8]. Other research groups have published recommendations for germline testing following TGT [6, 10, 11, 16–18]. These recommendations consider gene-specific factors such as germline conversion rate (GCR) and clinical actionability. GCR is the percentage of tumor-detected pathogenic variants confirmed as germline [4, 8]. Other recommendations include genetic variant-factors (e.g., founder mutations and pathogenicity interpretation in the germline), and individual patient-factors (e.g., age at diagnosis and relevant personal/family history of cancer or related phenotypes) [4, 6, 8, 10, 11, 16–18]. Despite this guidance, integrating germline genetic testing (GGT) recommendations for patients undergoing tumor-only sequencing remains challenging due to lack of expertise and poorly defined clinical pathways. Enhanced collaboration between oncologists and genetics clinics is crucial, particularly given the increasing use of larger tumor panels involving CSGs.

At the Princess Margaret Cancer Centre (PM), part of the University Health Network (UHN) in Toronto, Canada, a panel-based tumor testing program using next-generation sequencing (NGS) was established in 2016 as part of the Ontario-wide Cancer Targeted Nucleic Acid Evaluation (OCTANE) clinical trial [19]. This program focuses on characterizing advanced solid tumors using a 523-gene panel with DNA and RNA extracted from tumor tissues. As part of standard care activities [19], tumor genetic variants (TGVs) flagged as potentially germline are included in these reports, creating a growing need for systematic support in managing these findings. In response to this need, we developed a clinical pathway in 2022 at the PM Genetics clinic, based on ESMO and other relevant guidelines [4, 6, 8, 10, 11, 16–18], to assist medical oncologists in interpreting these unexpected findings and their hereditary cancer implications. This aggregate of guidelines was used to flag potential germline variants in tumor testing cases. Here, we provide an overview of our 1-year experience implementing this workflow.

## Methods

### OCTANE clinical trial enrollment

**Fig. 1:**
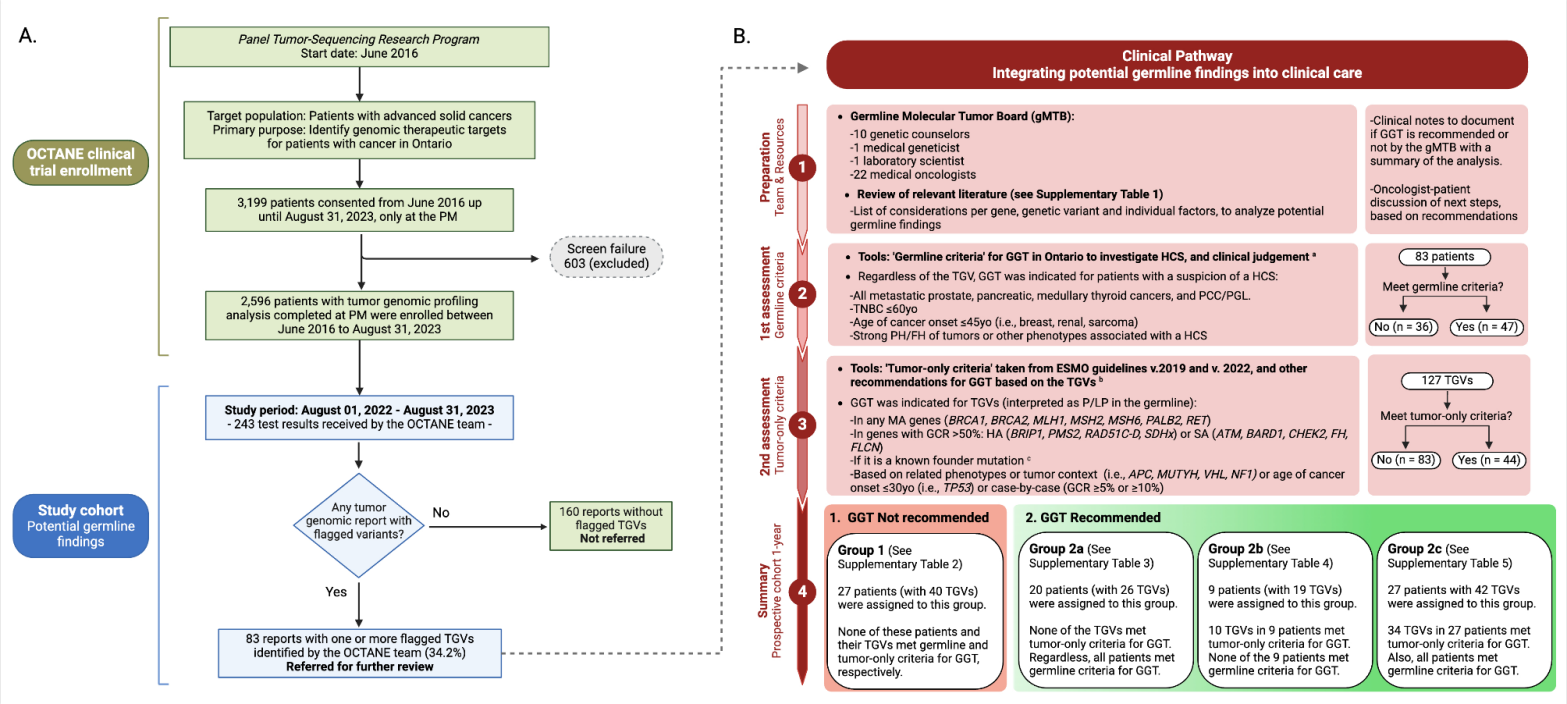
Workflow of the clinical pathway implemented at the Princess Margaret (PM) Cancer Centre to integrate potential germline findings from a tumor-sequencing research program, into clinical care (Created with BioRender.com). (**A**) Left panel shows the selection of tumor profiling reports that needed further review by the gMTB. (**B**) Right panel shows the steps taken for preparing the team and resources for the clinical pathway, as well as the following steps taken for the assessment of ‘germline and tumor-only criteria’, with a summary of the results. Cases were assigned to four different groups based on the recommendation for GGT and the reasons supporting it. **a**: Germline criteria, Cancer Care Ontario Hereditary Cancer Testing Eligibility Criteria [22]. A total of 83 patients were assessed according to ‘germline criteria’. **b**: Tumor-only criteria, recommendations for germline confirmation of TGVs as per tumor-only guidelines including The European Society of Medical Oncology (ESMO) Precision Medicine Working Group Germline Subgroup, and others [4, 6, 8, 10, 11, 16–18, 20, 21]. A total of 127 TGV were assessed according to ‘tumor-only criteria’. **c**: Founder mutations, a detailed list can be found in Methods section and Supplementary Material 1 - Tables 1 and 7 [6, 8, 9, 14, 22, 24, 27–29]. OCTANE, the Ontario-wide Cancer Targeted Nucleic Acid Evaluation (OCTANE) clinical trial (i.e., institutional research program) [19]; PM, Princess Margaret Cancer Centre; gMTB, germline molecular tumor board; GGT, germline genetic testing/confirmation; TGV, tumor genetic variants; HCS, hereditary cancer syndrome; PCC/PGL, pheochromocytomas/paragangliomas; TNBC, triple-negative breast cancer; PH/FH, personal history and/or family history; ESMO, the European Society of Medical Oncology; P/LP, pathogenic/likely pathogenic variants; GCR, germline conversion rate; MA, most-actionable; HA, high-actionability; SA, standard-actionability

The OCTANE clinical trial began in June 2016 and is currently ongoing [19]. The primary purpose of this large study is to identify genomic therapeutic targets for patients with advanced solid tumors from different cancer centres across Ontario. As of August 31, 2023, a total of 3,199 patients had been enrolled in this clinical trial at PM. The assessment of potential germline findings was integrated into the clinical trial protocol and informed consent process (UHN, Research Ethics Board: 16-5655, Ontario Cancer Research Ethics Board: 16–018), ensuring that patients were well-informed about the potential risks of these findings. Tumor genetic profiling was completed for 2,596 patients at the UHN Laboratory Medicine Program, Department of Pathology (Fig. 1A).

### Study cohort

The clinical pathway was implemented on August 1, 2022. Between then and August 31, 2023, the OCTANE study team received 243 tumor profiling results from cases analyzed consecutively by the UHN laboratory. The top five cancers sequenced were urinary tract (*n* = 44), endocrine (*n* = 43), breast (*n* = 28), colorectal (*n* = 28), and head and neck (*n* = 15). Of these, 83 (34.2%) had one or more TGVs flagged by the UHN laboratory as having potential germline significance. Following the clinical pathway (Fig. 1A), all reports with flagged TGVs were systematically referred to the gMTB for further evaluation. Details of the UHN laboratory’s criteria for flagging TGVs are provided in the Tumor Genomic Annotation section of the Methods.

### Internal clinical pathway preparation and implementation

A germline Molecular Tumor Board (gMTB) was formed, including up to 10 genetic counselors, one medical geneticist, a clinical laboratory scientist and 22 medical oncologists. We reviewed literature on germline-focused analysis of TGVs from society guidelines (e.g., ESMO v.2019 and v.2022) and other groups [4, 6, 8, 10, 11, 16–18, 20, 21], establishing key considerations per gene (e.g., germline conversion rate, clinical actionability), per genetic variant (e.g., founder mutations and pathogenicity interpretation in the germline), and individual patient-factors (e.g., age at diagnosis and family history of cancer) (Fig. 1B, Supplementary Material 1 - Table 1).

**Table 1:** Patient characteristics and assignation in each group based on the indication for germline confirmation

Characteristic		Groups after assessment by the gMTB				*p*-value
		GGT not recommended - Group 1	GGT recommended - Group 2			
	Total cases (*n* = 83)	Group 1 - Did not meet any criteria ‘germline and tumor-only’ (*n* = 27)	Group 2a - Only met ‘germline criteria’ (*n* = 20)	Group 2b - Only met ‘tumor-only criteria’ (*n* = 9)	Group 2c - Met both criteria ‘germline and tumor-only’ (*n* = 27)	
**Age at diagnosis**,** mean [SD]**^**a**^	55.5 [15.0]	61.1 [11.6]	58.6 [15.7]	55.1 [17.0]	47.7 [14.3]	**0.006**
**Sex**,** n (%)**^**b**^						
Female	41 (49.4%)	13 (48.1%)	9 (45.0%)	2 (22.2%)	17 (63.0%)	0.195
Male	42 (50.6%)	14 (51.9%)	11 (55.0%)	7 (77.8%)	10 (37.0%)	
**Cancer Type**,** n (%)**^**b**^						
Brain	2 (2.4%)	1 (3.7%)	0 (0.0%)	0 (0.0%)	1 (3.7%)	**0.006**
Breast	8 (9.6%)	1 (3.7%)	5 (25.0%)	0 (0.0%)	2 (7.4%)	
Colorectal	13 (15.7%)	10 (37.0%)	0 (0.0%)	1 (11.1%)	2 (7.4%)	
Melanoma	6 (7.2%)	1 (3.7%)	1 (5.0%)	2 (22.2%)	2 (7.4%)	
Endocrine	13 (15.7%)	1 (3.7%)	0 (0.0%)	1 (11.1%)	11 (40.7%)	
Urinary tract	20 (24.1%)	5 (18.5%)	9 (45.0%)	2 (22.2%)	4 (14.8%)	
Gynecological	3 (3.6%)	1 (3.7%)	2 (10.0%)	0 (0.0%)	0 (0.0%)	
Head & Neck	3 (3.6%)	3 (11.1%)	0 (0.0%)	0 (0.0%)	0 (0.0%)	
Pancreatobiliary	7 (8.4%)	1 (3.7%)	3 (15.0%)	1 (11.1%)	2 (7.4%)	
Sarcoma	2 (2.4%)	1 (3.7%)	0 (0.0%)	0 (0.0%)	1 (3.7%)	
Skin/Non-melanoma	2 (2.4%)	0 (0.0%)	0 (0.0%)	2 (22.2%)	0 (0.0%)	
Upper Gastrointestinal	4 (4.8%)	2 (7.4%)	0 (0.0%)	0 (0.0%)	2 (7.4%)	
**Completion of GGT (summary of cases)**,** n (%)**^**b, c**^						
Recommended - not performed	19 (34.0%)	NA	5 (25.0%)^d^	7 (77.8%)	7 (26.0%)^e^	**0.015**
Recommended - performed	37 (66.0%)	NA	15 (75.0%)	2 (22.2%)	20 (74.0%)	
**Description of GGT results for TGVs in 83 cases** ^**f**^	**Total TGVs** (***n* = 127**)	**Group 1 - Did not meet any criteria ‘germline and tumor-only’ ** **(40 TGVs from 27 cases)**	**Group 2a - Only met ‘germline criteria’ ** **(26 TGVs from 20 cases)**	**Group 2b - Only met ‘tumor-only criteria’ ** **(19 TGVs from 9 cases)**	**Group 2c - Met both criteria ‘germline and tumor-only’** **(42 TGVs from 27 cases)**	
GGT not recommended	83 TGVs	40 TGVs	26 TGVs	9 TGVs	8 TGVs	
GGT recommended	44 TGVs	0 TGVs	0 TGVs	10 TGVs (from 9 cases)	34 TGVs (from 27 cases)	
Confirmed somatic	18 TGVs	NA	NA	1 TGV (from 1 case)	17 TGVs (from 14 cases)	
Not performed (Unknown)	17 TGVs	NA	NA	8 TGVs (from 7 cases)	9 TGVs (from 7 cases)	
Confirmed germline	9 TGVs	NA	NA	1 TGV (from 1 case)	8 TGVs (from 8 cases)	

gMTB, germline molecular tumor board; GGT, germline genetic testing/confirmation; Germline criteria, patients met the Cancer Care Ontario Hereditary Cancer Testing Eligibility Criteria [22]; Tumor-only criteria, the TGV met the recommendation for germline confirmation as per tumor-only guidelines [4, 6, 8, 10, 11, 16–18]; SD, standard deviation; NA, not applicable. a: ANOVA test was used for age at diagnosis, since assumptions of normality and equal variances were verified with Shapiro-Wilk test (*p*-value 0.095; *p*-value stratified by groups > 0.1) and Levene’s test across groups (*p*-value 0.2). Pairwise comparisons using Tukey method with adjusted *p*-values are shown in a separate file (Supplementary Material 2 - Post Hoc test 1). b: Fisher-Freeman-Halton test was used to investigate for sex and cancer type differences. c: Fisher-Freeman-Halton test was used for cases with an indication of GGT (*n* = 56; Groups 2a to 2c) (Supplementary Material 1 - Tables 3, 4 and 5). d: Within Group 2a, 5/20 cases that met only ‘germline criteria’ did not have GGT before or after our recommendation (Supplementary Material 1 - Table 3). e: Within Group 2c, 7/27 cases received additional recommendation for GGT (mostly for a specific TGV or to extend a previous panel) but it was not performed (Supplementary Material 1 - Table 5). Moreover, two of these cases did not have any GGT done even though it was indicated as per ‘germline criteria’ and ‘tumor-only criteria’ (Supplementary Material 1 - Table 5). f: A total of 127 TGVs found in 83 cases were assessed by the gMTB. Only 44 TGVs from 36 cases (9 cases from Group 2b and 27 cases from Group 2c) were considered *germline relevant* and therefore recommended for GGT. A single case may have more than one TGV identified in their testing, leading to higher TGV counts than the total number of cases. Additionally, not all TGVs from cases in a specific group were *germline relevant* for GGT, which can explain the differences in totals across categories

As shown in Fig. 1B, patients were first assessed for germline testing on the basis of personal/family history of cancer, following the Cancer Care Ontario (CCO) Hereditary Cancer Testing Eligibility Criteria [22] and clinical judgment, referred to as ‘germline criteria’. Regardless of the TGV, all patients meeting ‘germline criteria’ received a recommendation for GGT. Some of these patients met ‘germline criteria’ based on their tumor type, regardless of age at diagnosis or family history (e.g., pancreatic cancer or metastatic prostate cancer). Other patients met ‘germline criteria’ due to their young age at diagnosis (e.g., ≤ 45 years for breast cancer and renal carcinoma) or the presence of specific subtypes (e.g., triple-negative breast cancer ≤ 60 years or chromophobe renal cancer). Patients with a strong personal or family history of tumors or other phenotypes associated with specific hereditary cancer syndromes (e.g., adenomatous polyposis or bilateral multiple lung cysts) were carefully documented to support the GGT recommendation.

The second assessment (Fig. 1B) evaluated the relevance of TGVs in the germline context, referred to as *germline relevant*. The UHN laboratory flagged potential germline variants (see Tumor Genomic Annotation) that were included in this assessment. *Germline relevant* TGVs were selected based on the considerations listed in the Supplementary Material 1 - Table 1 (referred to as ‘tumor-only criteria’) [4, 6, 8, 10, 11, 16–18, 20, 21]. Key factors for this assessment included gene actionability, TGV pathogenicity in the germline, and likelihood of germline origin (Fig. 1B). Founder mutations often found at a high frequency in genetically isolated populations due to shared ancestry, are typically of germline origin [23–26], therefore, these were prioritized for germline confirmation [23–25]. The list of founder mutations used as a source for the TGV assessment is detailed in Supplementary Material 1 - Tables 1 and 7 [6, 8, 9, 14, 22, 24, 27–29]. Other TGVs in specific genes were evaluated for germline testing based on tumor context, related phenotypes, and/or early onset (Fig. 1B). GGT and genetic counseling recommendations were documented in the patient’s Electronic Medical Record (EMR) and sent to their oncologist(s).

### Tumor genomic annotation

Details of sample collection, library preparation, and NGS using the Illumina TruSight™ Oncology 500 (TSO500) targeted hybrid-capture have been previously published [19]. The UHN laboratory generated variant calls using TSO500 LRM v2.2, aligned to the GRCh37/hg19 genome. TGV interpretation was based on Qiagen QCI platform (v7.1.20210428) results, and cross-referenced with databases like OncoKB, the Catalogue of Somatic Mutations in Cancer (COSMIC) [30, 31] and the biomedical literature, following the Association for Molecular Pathology, American Society of Clinical Oncology, and College of American Pathologists (AMP/ASCO/CAP) guidelines [32]. The UHN laboratory reported tiers I and II TGVs of strong and potential clinical significance, respectively, and tier III TGVs of unknown clinical significance but gain or loss of function (LOF). These interpretations were based on clinical significance in the somatic setting without determining somatic or germline origin because paired GGT was not performed. The UHN laboratory flagged TGVs in CSGs, following AMP/ASCO/CAP guidelines [32], indicating that further assessment is needed to recommend germline confirmation. This flag was applied to TGVs in CSGs with a variant allele fraction (VAF) of ≥ 20% in the tumor. According to ESMO tumor-only guidelines, this threshold reduces the number of flagged TGVs by 54% while retaining 96.5% of true germline variants [8]. Notably, *TP53* tumor variants were excluded from this flagging irrespective of VAF, owing to their higher likelihood of somatic origin. Variants descriptions were standardized according to Human Genome Variation Society (HGVS) recommendations, using the VariantValidator tool (https://variantvalidator.org/service/validate/batch/).

### Germline follow-up

Following the internal clinical pathway, the gMTB recommended either supporting or dissuading GGT to the oncologist as part of the clinical care of these 83 patients (Fig. 1B, Supplementary Material 1 - Table 1). If prior genetic testing was documented, these reports were retrieved to compare and decide if further testing was needed based on new personal or family history or on the TGV. If GGT was recommended, the oncologist discussed this with the patient. Upon obtaining patient’s consent, the oncologist facilitated GGT or referred the patient to the PM Genetics clinic for pretest genetic counseling. Patients with cancer and additional personal history of any hematological malignancy were offered testing on DNA from cultured fibroblasts obtained via skin-punch biopsy. For cases in which a hematologic malignancy was not involved, GGT was performed on DNA extracted from blood.

### Statistical analysis

Descriptive statistics were obtained for 83 patients, stratified based on gMTB recommendations for GGT following our internal workflow (Fig. 1B, Supplementary Material 1 - Table 1) [4, 6, 8, 10, 11, 16–18, 20–22]. Group 1 included cases where GGT was deemed unnecessary, as patients met neither ‘germline criteria’ nor ‘tumor-only criteria’. Group 2 comprised cases that fulfilled either ‘germline criteria’ (2a), or ‘tumor-only criteria’ (2b), or both (2c), warranting GGT in all three scenarios. Comparisons between these four groups evaluated differences in age at diagnosis, sex, cancer type, and completion rate of GGT. Characteristics of the 127 TGVs were also analyzed to compare *germline relevant* and not *germline relevant* TGVs. Categorical variables (sex, cancer type, gene actionability, variant class, somatic tier category, pathogenicity interpretation on the germline, and tumor-context) were described using frequency distributions. Statistical differences were assessed using the Fisher-Freeman Halton test for categorical variable comparisons between multiple groups with small cell counts (≤ 5 or zeros) [33], otherwise χ^2^ - test was used. Continuous variables (age at diagnosis, VAF, and GCR), were described using central tendency measures. Statistical assumptions were tested, and non-parametric alternatives were used if needed. The Shapiro-Wilk test assessed normality, and Levene’s test assessed variance homogeneity. Mean and standard deviation [SD] were used for age at diagnosis, and differences were assessed using ANOVA. Median and quartiles (Q) were used for VAF and GCR, and differences were assessed with Kruskal-Wallis rank-sum test. We considered as statistical significance a *p*-value ≤ 0.05. Pairwise post-hoc comparison using the Tukey method was performed for age at diagnosis. Pairwise post-hoc comparisons using Dunn’s test with Holm adjustments were conducted for VAF and GCR. The detection rate of true germline variants in this cohort of patients with advanced cancer is described. The statistical analyses were performed using the R Statistical Computing Software version 4.2.1 (2022-06-23) (R Foundation for Statistical Computing, Vienna, Austria).

## Results

### Patient characteristics

Potential germline variants were flagged in 34.2% (83/243) of OCTANE tumor profiling reports over a 1-year study period. The OCTANE study team referred these 83 reports for further review by the gMTB (Fig. 1A). The clinical characteristics of these cases are presented in Table 1. Mean age at diagnosis was 55.5 ± SD 15.0 years and 50.6% (*n* = 42) were male. The most common cancer primary sites were urinary tract (24.1%; *n* = 20), colorectal (15.7%; *n* = 13), endocrine (15.7%; *n* = 13), breast (9.6%; *n* = 8) and pancreatobiliary (8.4%; *n* = 7) (Table 1, Supplementary Material 1 - Fig. 1).

### First assessment – ‘germline criteria’

As shown in Fig. 1B, over half of the cases reviewed by the gMTB met ‘germline criteria’ for GGT (56.6%; 47/83), mainly due to a personal history of cancers requiring GGT, regardless of age at diagnosis or family history (e.g., pancreatic cancer) [22]. Many of these cases (74.5%; 35/47) had a prior documented visit to an Ontario genetics clinic for genetic cancer risk assessment. After this first assessment, the gMTB reviewed all tumor profiling reports from these 83 cases to determine which TGVs were *germline relevant* and required GGT (see the “Second assessment” section).

### Second assessment – ‘tumor-only criteria’

**Fig. 2:**
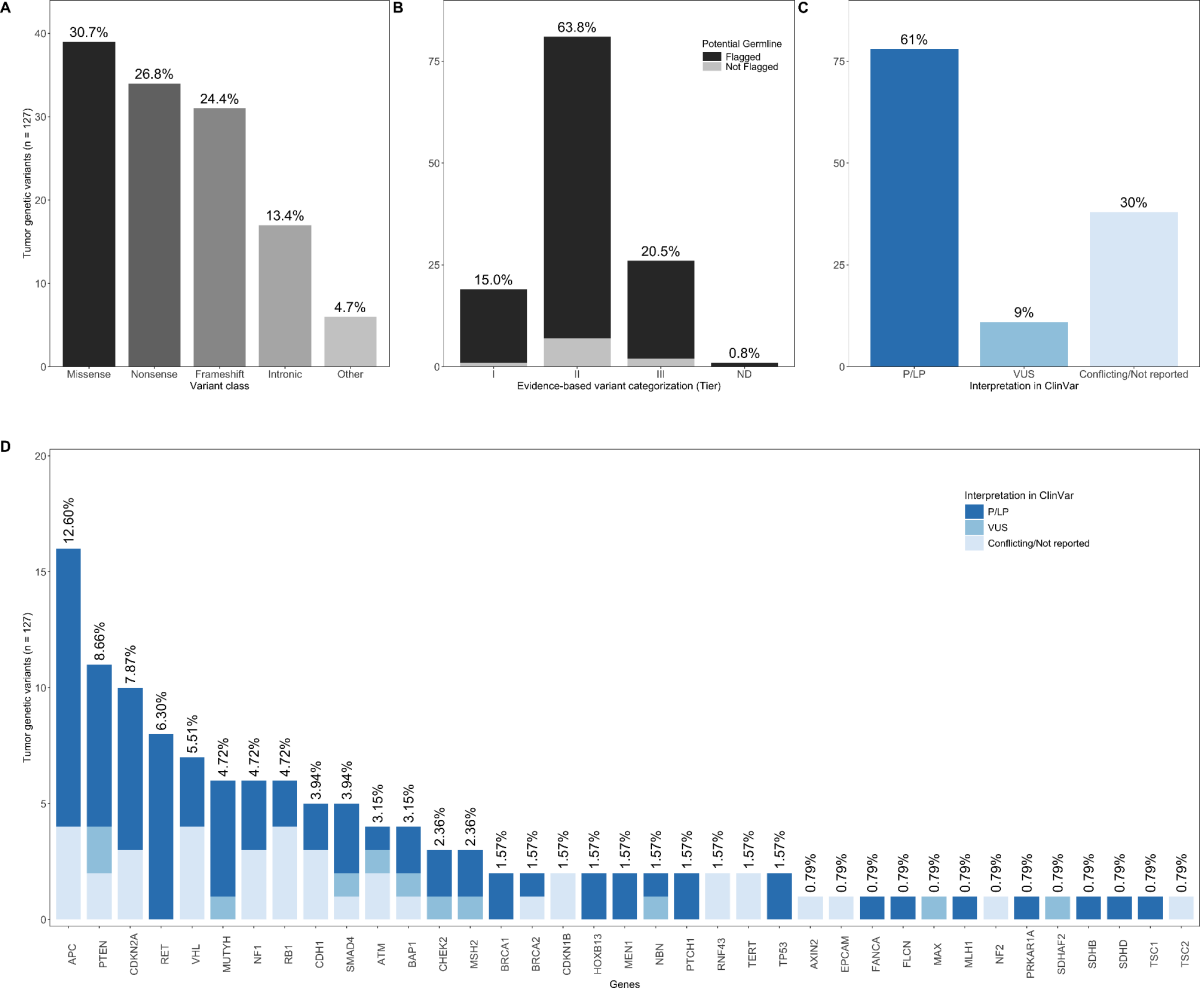
Description of 127 tumor genetic variants (TGV) reported for 83 cases. Top panels (A, B, and C): The Y-axis represents the number of assessed TGVs in each category: (**A**) Distribution of variant class category according to HGVS recommendations (https://hgvs-nomenclature.org/stable/). (**B**) Distribution of flagged variants according to the evidence-based variant category (Tier) assigned by the University Health Network (UHN) laboratory. (**C**) Distribution of the pathogenicity interpretation on the germline according to ClinVar [36]. Bottom panel: (**D**) Distribution of the pathogenicity interpretation on the germline according to ClinVar [36] categorized by gene for the 127 assessed TGVs. ND, not defined by the UHN laboratory; P/LP, pathogenic/likely pathogenic; VUS, variant of uncertain significance

A summary of the ‘tumor-only criteria’ for evaluating TGVs is shown in Fig. 1B. A list of considerations for this assessment was prepared in advance (Supplementary Material 1 - Table 1) [4, 6, 8, 10, 11, 16–18, 20, 21]. A total of 127 TGVs from 83 cases were assessed by the gMTB, as some tumors had multiple variants (1 TGV in 51 cases; 2 TGVs in 25 cases, 3 TGVs in 4 cases, 4 TGVs in 1 case and 5 TGVs in 2 cases) (Supplementary Material 1 - Tables 2, 3, 4 and 5). Most TGVs were LOF class (51.2%) and categorized as tier II (63.8%) in the somatic setting (Fig. 2A and B).

**Table 2:** Description of patients with confirmed P/LP germline variants

Age at diagnosis	Sex	Cancer type	Known germline *P*/LP variant			VAF in tumor genetic profiling	Personal history and phenotype	Relevant family history	Group assignation
			Yes/No	Gene	HGVS nomenclature				
50	Female	Adrenal cortical carcinoma	Yes	*FLCN*	NM_144997.7:c.694 C > T p.(Gln232Ter)	83%	Facial hirsutism; Two facial papules / angiofibromas; Bilateral renal cyst; Bilateral lung cyst	FDR: Melanoma (50) & Ovarian (54); SDR: Gastric (6), Bowel (50); TDR: Breast (*n* = 7)	Group 2c
57	Female	Thymoma	Yes	*RET*	NM_020975.6:c.2410G > A p.(Val804Met)	49%	Prophylactic thyroidectomy with medullary thyroid	FDR: Colon & Medullary thyroid (RET+), Papillary thyroid, Breast; SDR: Breast	Group 2c
29	Female	Thyroid cancer	No	*ATM*	NM_000051.4:c.7495G > T p.(Glu2499Ter)	48%	No	No	Group 2b
68	Male	Prostate cancer	Yes	*CHEK2*	NM_007194.4:c.470T > C p.(Ile157Thr)	47%	No	FDR: Prostate (*n* = 2)	Group 2c
32	Female	Breast cancer	Yes	*BRCA1*	NM_007294.4:c.5106del p.(Lys1702AsnfsTer4)	60%	Triple-negative breast cancer subtype, early onset	BRCA1+ (1 FDR, 2 SDR, 1 TDR); SDR: Breast (40s); TDR: Breast (63)	Group 2c
55	Male	Colorectal cancer	Yes	*CHEK2*	NM_007194.4:c.1427 C > T p.(Thr476Met)	36%	Bladder cancer (55)	FDR: Endometrial (58), Colorectum (68) SDR: Pancreatic (unk), Gastrointestinal (*n* = 3, 60–75), Prostate (60); TDR: Gastric (65s)	Group 2c
25	Male	Colorectal cancer	Yes	*MLH1*	NC_000003.12(NM_000249.4):c.1667 + 1G > T p.?	59%	MLH1/PMS2 absent, no MLH1 promotor hypermethylation; BRAF wild type (tumor profile)	FDR: Ovarian, Endometrial & Breast	Group 2c
33	Female	Diffuse high-grade glioma	Yes	*MSH2*	NM_000251.3:c.425 C > G p.(Ser142Ter)	76%	MSH2/MSH6 absent (tumor profile)	FDR: Prostate (30s) & Colon (50s); SDR: Cervix (40s); Colon (85)	Group 2c
35	Female	Breast cancer	Yes	*BRCA2*	NM_000059.4:c.5576_5579del p.(Ile1859LysfsTer3)	83%	Breast cancer, early onset	No	Group 2c

P/LP, pathogenic/likely pathogenic; HGVS, Human Genome Variation Society; VAF, variant allele fraction; FDR, first degree relative; SDR, second degree relative; TDR, third degree relative; Unk, unknown. a: Case-report published by Hofstedter R, et al. 2023 [48]

Of the 127 TGVs assessed, 117 were flagged as potential germline variants by the UHN laboratory. Previous reports indicate a wide range of VAF in tumor sequencing for confirmed somatic and germline variants [18, 34, 35]. Based on this, additional 10 TGVs not flagged in these reports by the laboratory (Fig. 2B) but involving actionable CSGs (defined in ESMO guidelines) [4, 8] at VAFs < 20%, or found in the *TP53* gene in early-onset cancer cases (≤ 30 years), were included for further assessment by the gMTB. Most TGVs were interpreted as pathogenic/likely pathogenic (P/LP) in the germline, accounting for 61.4% (78/127), whereas 30% (38/127) had conflicting interpretations or were not reported in ClinVar [36] (Fig. 2C).

**Fig. 3:**
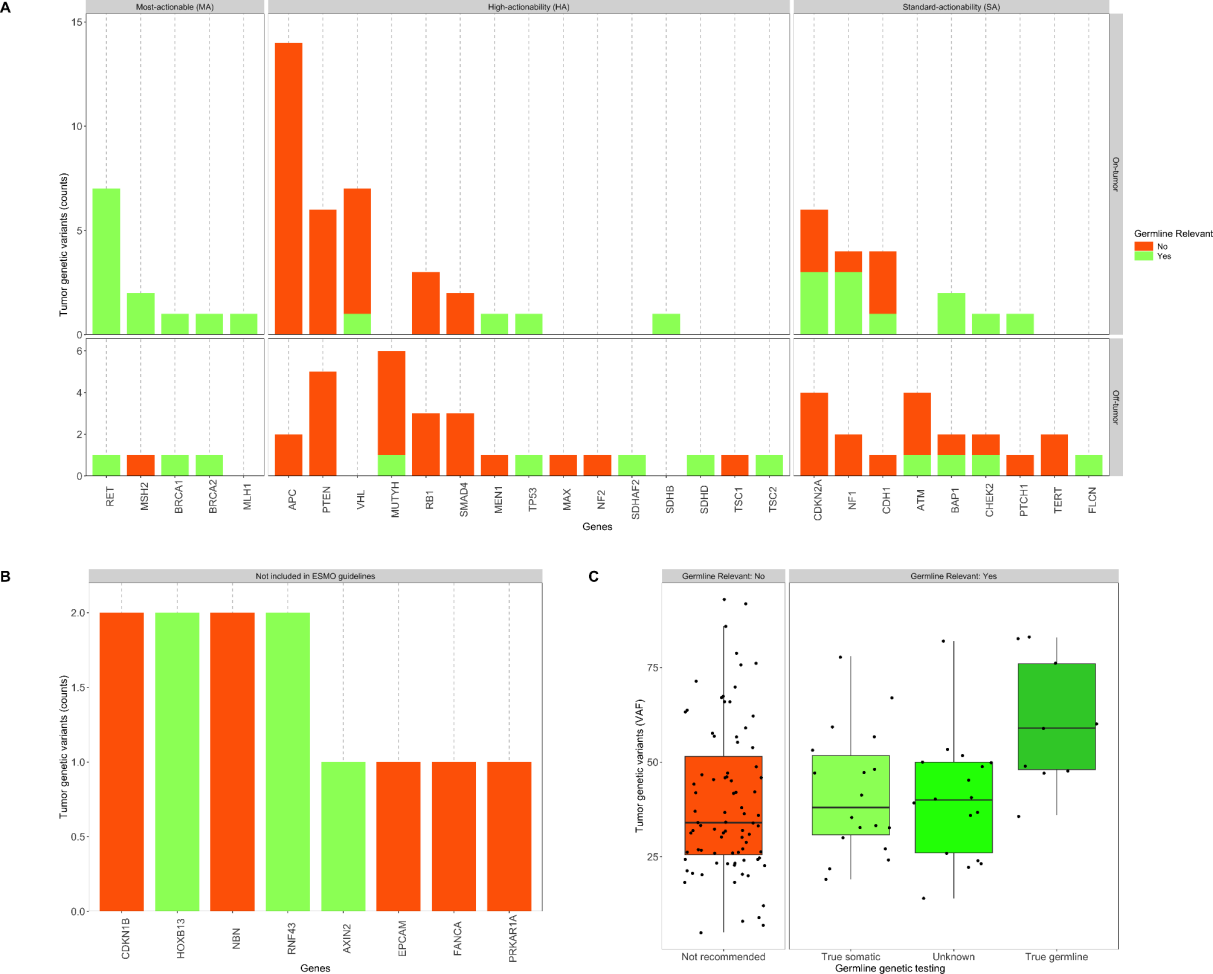
Description of the 37 genes with tumor genetic variants (TGV) reported for 83 cases, according to their actionability as per ESMO guidelines [4, 8]. (**A**) Genes categorized as most-, high- and standard-actionable with TGV considered *germline relevant* (green) or not (red), in on-tumor (top panel) and off-tumor (bottom-panel) context. (**B**) Genes not included in ESMO guidelines [4, 8] with TGV flagged as potential germline, that the gMTB considered *germline relevant* (green) or not (red). (**C**) Boxplot of the VAF distribution among TGV not recommended for germline confirmation (red) and those considered *germline relevant* (green gradient) that needed confirmation. Germline confirmation results: “True somatic” variants (light green), “Unknown” are those with genetic testing pending or not done (medium green), and “True germline” variants (dark green). MA, most-actionable; HA, high-actionability; SA, standard-actionability; ESMO, European Society for Medical Oncology; VAF, variant allele fraction

A total of 37 genes with ≥ 1 TGV were analyzed (Supplementary Material 1 - Table 6). The distribution of variants per gene and pathogenicity interpretation are shown in Fig. 2D. Gene actionability was assigned according to the ESMO guidelines [4, 8, 37] (Fig. 3A, Supplementary Material 1 - Table 6). Most TGVs were detected in high-actionability genes (48.8%; 62/127), followed by standard-actionability genes (29.1%; 37/127) and most-actionable genes (12.6%; 16/127) (Supplementary Material 1 - Table 6). The remaining TGVs assessed by the gMTB were in 8 CSGs not included in the ESMO guidelines and flagged as potentially germline by the UHN laboratory (9.5%; 12/127) (Fig. 3B, Supplementary Material 1 - Table 6). Known founder mutations [6, 8, 9, 14, 22, 24, 27–29] were identified in tumor profiling reports of seven unique cases (8.4%; 7/83), including *CHEK2* (I157T, *n* = 1), *HOXB13* (G84E, *n* = 2) and *MUTYH* (G368D, *n* = 2 and Y151C, *n* = 2) (Supplementary Material 1 - Tables 6 and 7).

After applying ‘tumor-only criteria’ to the 127 TGVs, we identified 44 TGVs as *germline relevant*, warranting GGT recommendations. The remaining 83 TGVs were considered *not germline relevant* (Figs. 1B and 3A and B, Supplementary Material 1 - Tables 6 and 8). Differences between groups (*germline relevant* and *not germline relevant*) are shown in Supplementary Material 1 - Table 8. Most TGVs categorized by the UHN laboratory as tier I were selected for germline confirmation (68.4%; 13/19); whereas fewer tier II (30.9%; 25/81) and tier III TGVs (23.1%; 6/26) were selected (*p*-value 0.003). While pathogenicity is important for germline confirmation, only 42.3% of all TGVs interpreted in the germline as P/LP were selected as *germline relevant* (*p*-value 0.045). Most *germline relevant* TGVs were found in most- and standard- actionable genes, mainly in relation to the high GCR reported for several of these genes (*p*-value 0.001). The median GCR of TGVs in any tumor context was significantly different between groups, with higher rates in the group of *germline relevant* (on-tumor 22.0%, Q1-Q3: 6.7–39.1; and off-tumor 78.7%, Q1-Q3: 38.5–88.7) compared to the *not germline relevant* group (on-tumor 0.7%, Q1-Q3: 0.7–3.6; and off-tumor 4.5%, Q1-Q3: 1.2–45.4) (*p*-value ≤ 0.001). All pairwise comparisons between groups were significantly different (adjusted *p*-values < 0.01), except for *germline relevant* [off-tumor versus on-tumor] and *not germline relevant* [off-tumor] versus *germline relevant* [on-tumor] (adjusted *p*-values > 0.1) (Supplementary Material 2 - Post Hoc test 2).

### Comparison of clinical characteristics among groups

Based on gMTB recommendations either supporting or dissuading GGT, cases were assigned to groups (Fig. 1B; Table 1). GGT was not recommended in 32.5% (27/83) of the cases (Group 1) (Fig. 1B; Table 1, Supplementary Material 1 - Table 2). Conversely, all remaining cases had indication for GGT (Group 2: 67.5%, 56/83) (Fig. 1B; Table 1). Additional subgroups were created stemming from the reasons supporting the GGT recommendation. Cases meeting only ‘germline criteria’ were assigned to Group 2a (35.7%, 20/56) (Supplementary Material 1 - Table 3), cases meeting solely ‘tumor-only criteria’ were assigned to Group 2b (16.1%, 9/56) (Supplementary Material 1 - Table 4), and cases meeting both ‘germline and tumor-only criteria’ were assigned to Group 2c (48.1%, 27/56) (Supplementary Material 1 - Table 5).

Significant differences in mean age at diagnosis were observed among groups (*p*-value 0.006) (Table 1). Group 2c cases had a lower mean age (47.7 ± SD 14.3 years) compared with those in Group 1 (61.1 ± SD 11.6 years) and Group 2a (58.6 ± SD 15.7 years) (adjusted *p*-value ≤ 0.05) (Supplementary Material 2 - Post Hoc test 1). Cancer type distribution showed significant differences between groups (*p*-value 0.006) (Table 1), with relevance to cancer types that meet the ‘germline criteria’ in Groups 2a and 2c, including metastatic prostate (within the urinary tract disease site), breast, pancreatic, and endocrine cancers (Supplementary Material 1 - Fig. 2). In contrast, Groups 1 and 2b, which do not meet these criteria, exhibit a more diverse array of cancer types (Table 1, Supplementary Material 1 - Fig. 2). Some cancer types showed more differences in their distribution across groups. For example, most colorectal cancers were assigned to Group 1 (76.9%; 10/13), while fewer cases (23.1%; 3/13) were assigned to Group 2 (1/13 in Group 2b and 2/13 in Group 2c) (Supplementary Material 1 - Fig. 2). In contrast, 92.3% (12/13) of endocrine cancers, mostly medullar thyroid carcinoma and pheochromocytomas/paragangliomas, were assigned to Group 2 (1/13 in Group 2b and 11/13 in Group 2c), while only 1/13 (7.7%) was assigned to Group 1 (Supplementary Material 1 - Fig. 2). For the urinary tract disease site, which the majority consisted in metastatic prostate cancer and renal cell carcinomas, and to a lesser extend other sites (i.e., bladder and urothelial), 75% (15/20) were assigned to Group 2 (9/20 in Group 2a, 2/20 in Group 2b, and 4/20 in Group 2c), while 25% (5/20) were assigned to Group 1 (Supplementary Material 1 - Fig. 2).

None of the 27 (100%; 27/27) patients in Group 1 had documentation of GGT performed before or after tumor testing (Supplementary Material 1 - Table 2). Among Group 2 cases, 62.5% (35/56) had undergone GGT prior to tumor testing (12/20 cases in Group 2a, 0/9 cases in Group 2b, and 23/27 cases in Group 2c) (Supplementary Material 1 - Tables 3, 4 and 5). Overall counts of cases recommended for GGT by the gMTB (despite any genetic testing done before) for which genetic testing was not completed (i.e., not performed), are shown in Table 1. Compliance with GGT recommendations was influenced by the patient’s survival status, clinician’s judgment, and patient’s preferences (Supplementary Material 1 - Table 9). In Group 2b, the majority of GGTs recommended by the gMTB were not completed, resulting in a completion rate of 22.2% (2/9), the lowest among groups. In contrast, Groups 2a and 2c, which included patients with suspected hereditary cancer syndrome had higher completion rates of 75.0% (15/20) and 74.0% (20/27), respectively. The completion rates of genetic tests showed significant differences between groups (*p*-value 0.015) (Table 1).

### Confirmation of P/LP variants in the germline

In this study, 9 cases had TGVs confirmed as P/LP in the germline (Tables 1 and 2), representing 24.3% (9/37) of those who completed GGT for any reason (*n* = 37; 15/20 in Group 2a, 2/7 in Group 2b, and 20/27 in Group 2c) (Table 1). The remaining 46/83 cases in our cohort did not have genetic testing (27/27 in Group 1 did not meet any indication, and 19/56 in Group 2 with indication for GGT but unable to be performed). Overall, these 9 cases with confirmed P/LP variants in the germline account for 10.8% (9/83) of the entire cohort assessed by the gMTB. GGT was completed for only 27 of the 44 *germline relevant* TGVs, resulting in a GCR of 33.3% (9/27). Most confirmed germline variants (8/9) were found in Group 2c and were linked to personal or family history. These patients had undergone GGT prior to tumor testing, which flagged these variants. We identified only one germline-confirmed tumor variant in a Group 2b case, where the patient underwent GGT following the gMTB recommendation. Two P/LP variants were detected in *CHEK2*, and other mutated genes were *FLCN*,* RET*,* ATM*,* BRCA1*,* MLH1*,* MSH2* and *BRCA2*, each with one variant (Table 2). The median VAF was 59.0% [Q1-Q3: 48.0–76.0] for TGVs confirmed as germline, 38.0% [Q1-Q3: 30.8–51.8] for TGVs confirmed as somatic, and 40.0% [Q1-Q3: 26.0–50.0] for TGVs with unknown germline results (Fig. 3C, Supplementary Material 1 - Table 8). TGVs not recommended for GGT had a median VAF of 34.0% [Q1-Q3: 25.5–51.5], which was significantly lower than those confirmed as germline (*p*-value 0.021; adjusted *p*-value 0.011) (Supplementary Material 2 - Post Hoc test 3).

## Discussion

Our study shows that 34.2% of tumor profiling tests in patients with advanced cancer reveal potential germline findings. A study of 710 patients with advanced cancer from Japan revealed a similar 40% rate of potential germline findings in 46 CSGs [38]. A larger study of 125,128 cases reported a lower rate (9.7%) of potential germline findings, after analyzing only 24 CSGs known to have a high GCR (> 10%) [8, 14]. This highlights the impact of gene selection and filtering methods on reported rates of potential germline findings.

Following the ‘germline criteria’, over half of the cases assessed by the gMTB were recommended for GGT, irrespective of tumor findings (47/83). The most common criteria included personal history of metastatic prostate cancer (*n* = 8), medullary thyroid cancer (*n* = 7), pancreatic cancer (*n* = 6), and breast cancer ≤ 45 years (*n* = 3). Surprisingly, 25.5% of these cases (12/47) lacked evidence of prior visits to Ontario genetics clinics for genetic cancer risk assessments. Underutilization of GGT in patients with cancer meeting ‘germline criteria’ has been documented [25, 39], with older and racially diverse patients less likely to receive GGT [39]. As germline-directed cancer screenings, risk-reducing surgeries and targeted therapies can improve survival, integrating tumor molecular profiling into clinical practice is essential for identifying more cases requiring GGT [25, 39]. Consequently, after second assessment based on ‘tumor-only criteria’, 57.4% (27/47) of these cases were identified with *germline relevant* TGVs that prompted GGT (Group 2c), and for some of these it required additional studies beyond the hereditary cancer syndrome that was under investigation.

Among cases not meeting ‘germline criteria’ for GGT (36/83), 27 were assigned to Group 1 because none of the TGVs were considered *germline relevant* by the gMTB after the second assessment based on ‘tumor-only criteria’. In contrast, the gMTB identified 10 *germline relevant* TGVs in 25.0% (9/36) of patients not meeting ‘germline criteria’, which were assigned to Group 2b. These cases would have otherwise gone undetected had it not been for tumor testing and the expertise of the gMTB. Within Group 2b cases, we confirmed the origin of two relevant TGVs: one germline *ATM* variant in a patient with papillary thyroid cancer and one somatic *BAP1* variant in a patient with renal cell carcinoma. Despite the gMTB’s recommendation to pursue GGT, testing was not completed for other *germline relevant* TGVs in this group. Among these were three variants with a high likelihood of germline origin, independent of VAF [40], identified in two patients who passed away before undergoing GGT: a *BRCA1* TGV (VAF 37%) in a bladder cancer, and a *BRCA2* TGV (VAF 14%) along with the *HOXB13 G84E* founder mutation (VAF 53%) in a porocarcinoma.

We employed expert-led pathways to guide clinical interpretation and GGT decisions, following ASCO guidelines for communicating medically relevant potential germline variants identified through tumor sequencing to patients in clinical settings [41]. We recognized that VAF in tumors alone cannot definitively confirm or exclude a germline origin [25], as tumor purity and concurrent somatic events can impact VAF, making it an unreliable indicator for determining germline variants [25, 35, 40]. Considering reports showing wide tumor VAF ranges between somatic and germline variants [18, 34, 35], the gMTB evaluated on a case-by-case basis tumor variants in CSGs not flagged by the UHN laboratory due to VAFs below the 20% threshold. However, in our study, none of the TGVs with VAF < 20% that received recommendation for germline confirmation and underwent GGT were of germline origin.

Overall, 34.6% (44/127) of the TGVs were identified as *germline relevant*, warranting GGT recommendations based on key features. Nearly 70% of TGVs categorized as tier I in the somatic setting were deemed *germline relevant*. Pathogenicity interpretation in the germline setting was an important feature, although less than 50% of P/LP variants were considered *germline relevant*, showing that this alone was not a deciding factor for germline confirmation. In accordance, TGVs located in most- or standard-actionable genes accounted for most *germline relevant* variants (30/44), many of which have a GCR ≥50% in any tumor and age context (63%; 19/30): *ATM*,* BRCA1*, *BRCA2*, *CHEK2*,* FLCN*,* MLH1*, *MSH2*, and *RET*. Clinical judgment due to related phenotypes (*AXIN2*,* CDH1*,* MEN1*, *MUTYH*, *NF1*, *RNF43*, and *VHL*), was an important factor for recommending GGT testing, regardless of VAF or GCR. Finally, GGT was recommended for founder mutations, such as those in *HOXB13* and *CHEK2*, since these are most likely germline [23–25]. While ESMO guidelines state that *MUTYH* should be included in germline-focused analysis, recommendations for reporting and follow-up testing are limited upon detecting two pathogenic variants in that gene due to its association with the autosomal recessive condition, MAP (*MUTYH*-Associated Polyposis) [4, 8]. None of our cases with *MUTYH* TGVs met this criterion; thus, any founder *MUTYH* TGV, was communicated to the treating oncologist to discuss the relevance for family members, and for only one case the gMTB recommended germline confirmation given a clinical suspicion of MAP syndrome in the family.

In our study, GGT was completed for 27 of the 44 *germline relevant* TGVs. Among these, 9 TGVs were confirmed to be germline, resulting in a GCR of 33.3% (9/27). These 9 TGVs were detected in 9 cases from our study cohort, representing 24.3% of all those who completed GGT for any reason (*n* = 37). Accounting for the entire cohort assessed for germline testing eligibility following our clinical pathway, these 9 cases with confirmed P/LP variants in the germline represent 10.8% (9/83). Many studies have reported rates of clinically actionable inherited variants ranging from 4.3 to 17.5% [24, 42–46]. Recently, a similar study that evaluated the clinical utility of molecular tumor boards guiding recommendations for GGT based on TGVs assessment, reported an overall GCR of 42.5% [46], in line with our study. Our rate of germline P/LP carriers identified through tumor sequencing approach that would have been missed otherwise (11.1%, 1/9 in Group 2b), is comparable to previous reports of 9.7% [24]. The higher confirmation of P/LP germline variants in Group 2c reflects stronger combined clinical and genomic evidence, as well as higher rates of prior genetic testing compared to Group 2b, providing additional insights into these cases. Interestingly, Group 2a (‘germline criteria’ only without *germline relevant* TGVs) did not identify any germline variants, suggesting negative tumor testing may indicate the absence of a germline variant. Still, these cases need to be investigated for a hereditary cancer syndrome and would require germline testing regardless of their tumor findings [25].

Many cancer centres face resource limitations that hinder comprehensive evaluations using paired tumor-normal sequencing, with most publications stemming from research [4, 8, 24, 25]. While tumor genomic sequencing is increasingly used in clinical practice, oncologists lack a clear pathway for interpreting potential germline findings. Our study integrates tumor-sequencing research into clinical practice, addressing gaps like missed carriers due to testing criteria and the underuse of genetic testing in eligible patients. This model bridges tumor-sequencing research and clinical-grade germline genetics through a multidisciplinary process that identifies TGVs requiring germline confirmation. We highlight the essential role of genetic counselors in interpreting tumor testing results through the gMTB and emphasize the value of clinical judgment and tumor-specific criteria in cancer risk assessment. Moreover, we present a list of key considerations [4, 6, 8, 10, 11, 16–18, 20, 21] and founder mutations [6, 8, 9, 14, 22, 24, 27–29] relevant to germline-focused TGV analysis. This compilation aligns with international guidelines from ESMO [4, 8], ASCO [25], and NCCN [47], further supporting the real-world integration of these findings into clinical practice.

This single- institution study with a small sample size has limitations that may affect the extrapolation of our findings. Changes in enrollment patterns since the OCTANE trial began may have affected the types of cancers analyzed. For example, the number of advanced breast cancer cases sequenced during our study period decreased compared to previous years, while the number of advanced urinary tract cancer cases sequenced remained consistent. These shifts may have led to an overrepresentation of certain tumor types in our cohort. Consequently, our findings may not be fully generalizable to other tumor types or clinical settings and should be considered when interpreting the conclusions drawn from this study. The gMTB relied on flagging by the UHN lab, meaning that reports with only TGVs with VAF < 20%, were not flagged/referred. To understand the effect of this, we reviewed the remaining 160 non-referred reports and identified five cases where the gMTB would have recommended GGT according to ‘tumor-only criteria’. These were not included in our statistical analysis given that two patients had prior negative GGT and three were lost to follow-up. Additionally, 24.4% (39/160) of the non-referred cases fulfilled ‘germline criteria’. Among these, 82.1% (32/39) were referred to GC and underwent GGT, all with negative results. Of the remaining cases, six did not undergo GC/GGT (four were not referred and two declined). One additional case involved a patient referred to GC but passed away before testing. Changes in ESMO guidelines (2019 vs. 2022) [4, 8], led to differing recommendations for *ATM* and *CDKN2A* TGVs. Incomplete GGT limited the analysis of cases with *germline relevant* TGVs not meeting ‘germline criteria’. Finally, tumor annotation criteria are heterogeneous and traditionally used to guide cancer treatment rather than to identify germline variants, which may have led to missed variants with potential germline relevance. To address this, we employed multiple annotation approaches. Initiatives such as Clinical Interpretation of Variants in Cancer (CIViC; https://civicdb.org/welcome) and Clinical Variant (ClinVar; https://www.ncbi.nlm.nih.gov/clinvar/), aim to standardize these methods, which will be instrumental in improving the identification of germline variants in tumor sequencing.

## Conclusions

Identifying patients who benefit from germline confirmation of TGVs requires combining clinical judgment with tumor- and gene-specific criteria. Developing an up-to-date clinical pathway was crucial for integrating germline genetic evaluation into tumor profiling at the PM.

## Electronic supplementary material

Below is the link to the electronic supplementary material.

Supplementary Material 1

Supplementary Material 2

## Data Availability

The datasets used and/or analysed during the current study are available from the corresponding author on reasonable request.

## Abbreviations

AMP/ASCO/CAP: Association for molecular pathology, american society of clinical oncology, and college of american pathologists
CCO: Cancer care ontario hereditary cancer testing eligibility criteria or germline criteria
CSGs: Cancer susceptibility genes
EMR: Electronic medical record
ESMO: European society of medical oncology precision medicine working group germline subgroup or tumor-only criteria
FFPE: Formalin-fixed, paraffin-embedded
GCR: Germline conversion rate
GGT: Germline genetic testing
gMTB: Germline molecular tumor board
HCS: Hereditary cancer syndrome
HA: High-actionability gene
LOF: Loss of function
MA: Most-actionable gene
NGS: Next-generation sequencing
OCTANE: Ontario-wide cancer targeted nucleic acid evaluation clinical trial
P/LP: Pathogenic or likely pathogenic
PM: Princess margaret cancer centre
SD: Standard deviation
SA: Standard-actionability gene
TGT: Tumor genetic testing
TGVs: Tumor genetic variants
UHN: University health network
VAF: Variant allele fraction
VUS: Variants of uncertain significance
