# Supplementary material for: Clinical integration of germline findings from a tumor testing precision medicine program
Source: BMC Cancer. 2025 Jan 30;25:176. doi: 10.1186/s12885-025-13487-4 (PMC11783960; doi:10.1186/s12885-025-13487-4)
Supplement: Supplementary file 1 — Supplementary Material 1 [file 12885_2025_13487_MOESM1_ESM.pdf]

Supplementary Material 1

Supplementary Tables and Figures

Supplementary Table 1. Review of the considerations for analyzing potential germline findings from OCTANE reports to include in the internal workflow and generate recommendations on genetic counseling or testing (starting date August 01, 2022)

Variable	Criteria - questions	Considerations	Supporting literature
**Gene**	1. Is the gene clinically important?	1.1. High risk genes with clear management recommendations<br>1.2. Well-defined risk with management recommendations<br>1.3. Poorly characterized or without clear recommendations	[1, 2]
	2. Reported germline/overall mutation ratio	2.1. Germline/overall mutation ratio is >0.25<br>2.2. Germline/overall mutation ratio is <0.25<br>2.3. Not reported	[3]
	3. Reported germline conversion rate (GCR) >10%[a]	3.1. Germline conversion rate >10% on-tumor<br>3.2. Germline conversion rate >10% off-tumor<br>3.3. Updated information on germline conversion rate (threshold of >5% or >10%)<br>3.4. None of the above or not reported	[1, 4]
**Genetic variant**	4. Is a known or reported genetic variant (i.e., Clinvar, ClinGen)?<br>Note: irrespective of the Variant Allele Fraction (VAF)[b]	4.1. Well characterized founder mutation[c]<br>4.2. Well characterized disease-causing variant associated with a Hereditary Cancer Syndrome<br>4.3. Well characterized and interpreted as non-causing disease<br>4.4. Not well characterized but highly expected to cause disease or alteration of the gene function (i.e., Loss Of Function, LOF)<br>4.5. Needs the application of an automated tools for variant curation	[1-3, 5-8]
**Individual**	5. Age at diagnosis is suspicious[d]	5. Age at diagnosis is earlier than expected for that type of tumor?	[1, 6, 9, 10]
	6. Phenotype is coherent with the syndrome associated with the mutated gene?[d]	6.1. Tumor type is within the spectrum of the syndrome associated to that gene?<br>6.2. Present other manifestations of the syndrome associated to that gene?	[1, 6, 9, 10]
	7. Family history is suspicious[d,e]	7.1. Strong family history coherent with the syndrome associated to that gene?<br>7.2. Other familial characteristics and need further genetic counseling<br>7.3. Without relevant family history	[1, 6, 9, 10]

OCTANE, the Ontario-wide Cancer Targeted Nucleic Acid Evaluation (OCTANE) clinical trial [11].

a: Updated germline conversion rates and thresholds were published later (Dec 16, 2022) by the European Society for Medical Oncology (ESMO) Precision Medicine Working Group [4].

b: A study found that 22% of germline variants detected at a VAF between 40% to 60% in the normal sample, had a VAF in the paired tumor sample outside of this range: either at a higher VAF (10% with VAF >60%) or a lower VAF (11% with VAF <40%, some even below VAF 20%) [12]. Given this and other studies showing the overlapping VAF ranges in tumor sequencing obtained for confirmed somatic and germline variants [8, 12, 13], the gMTB decided to analyze all TGVs in these reports regarding their relevance on the germline (flagged and not flagged by the UHN laboratory). For example, TGVs with VAF <20% in any actionable CSG with a high GCR (regardless of the tumor context, like *BRCA1*), or in a CSG in a patient with phenotypes related to that gene and/or early-onset (like *TP53* in a sarcoma case diagnosed <30 years, regardless of VAF), should be considered for germline confirmation.

c: Founder mutations considered for germline confirmation included: one in *APC*: NM_000038.6:c.3920T>A p.(Ile1307Lys); one in *ATM*: NM_000051.4:c.7271T>G p.(Val2424Gly); two in *BRCA1*: NM_007294.4:c.68_69del p.(Glu23ValfsTer17) and NM_007294.4:c.5266dup p.(Gln1756ProfsTer74); one in *BRCA2*: NM_000059.4:c.5946del p.(Ser1982ArgfsTer22); four in *CHEK2*:

NM_007194.4:c.1100del p.(Thr367MetfsTer15), NC_000022.11(NM_007194.4):c.444+1G>A p.?, NM_007194.4:c.470T>C p.(Ile157Thr), and NM_007194.4:c.1283C>T p.(Ser428Phe); one in *HOXB13:* NM_006361.6:c.251G>A p.(Gly84Glu); one in *MSH2:* NM_000251.3:c.1906G>C p.(Ala636Pro); two in *MSH6*: NM_000179.3:c.3984_3987dup p.(Leu1330ValfsTer12) and NM_000179.3:c.3959_3962del p.(Ala1320GlufsTer6); two in *MUTYH*: NM_001048174.2:c.452A>G p.(Tyr151Cys) and NM_001048174.2:c.1103G>A p.(Gly368Asp); and one in *TP53*: NM_000546.6:c.1010G>A p.(Arg337His) [1, 3, 14-20].

d: Any patient that meets the Cancer Care Ontario Hereditary Cancer Testing Eligibility Criteria [14] should be referred to genetic counseling, regardless of tumor genetic findings.

e: Such as: unknown or limited family structure, both sides of the family with cancer cases, Ashkenazi ancestry, consanguineous parents.

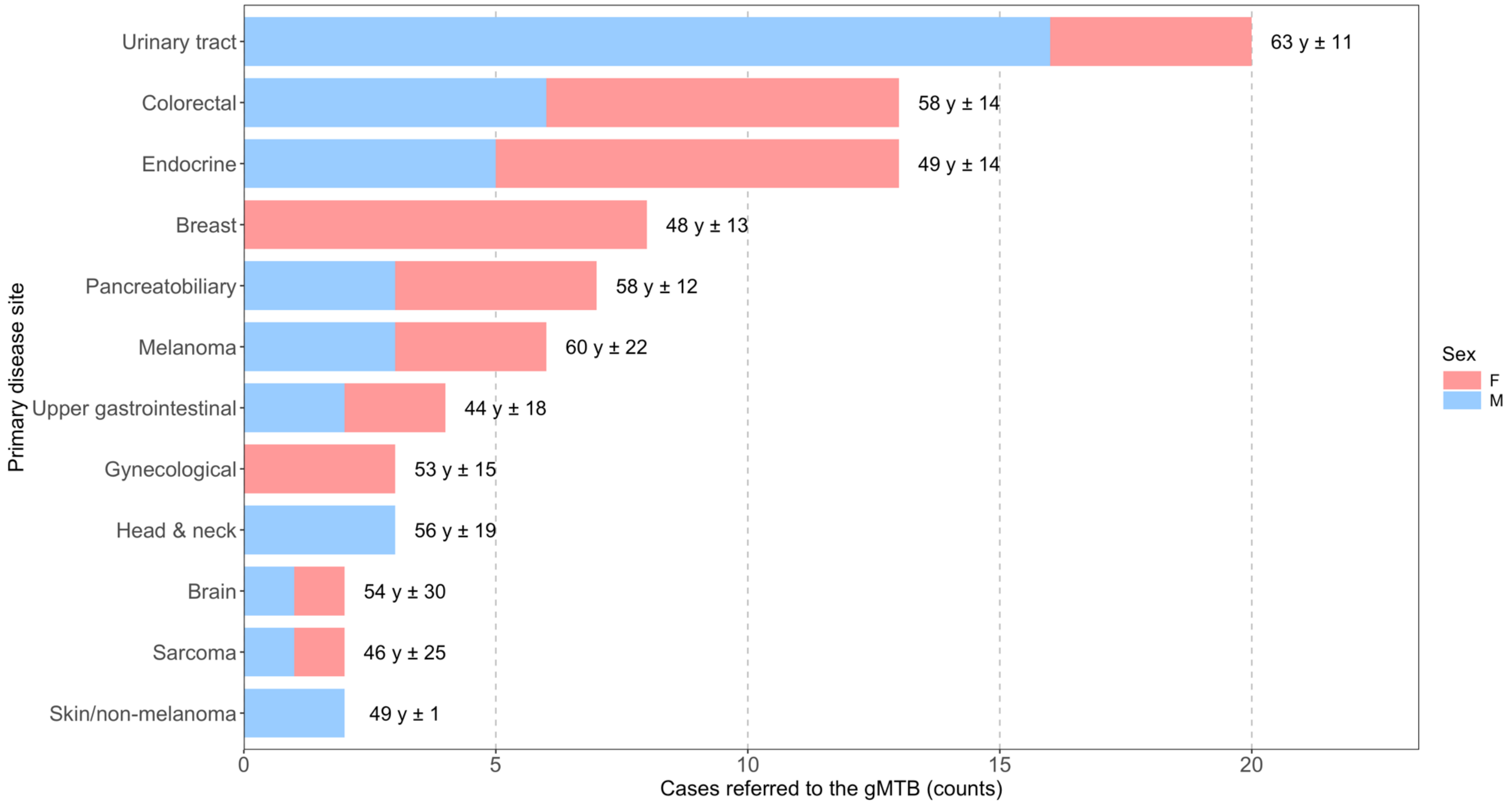

Supplementary Fig. 1 Distribution of 83 cases referred to the gMTB by cancer site and sex. Mean age at diagnosis per cancer and SD is shown at the right side of each bar. gMTB, germline molecular tumor board; F, female; M, male; SD, standard deviation.

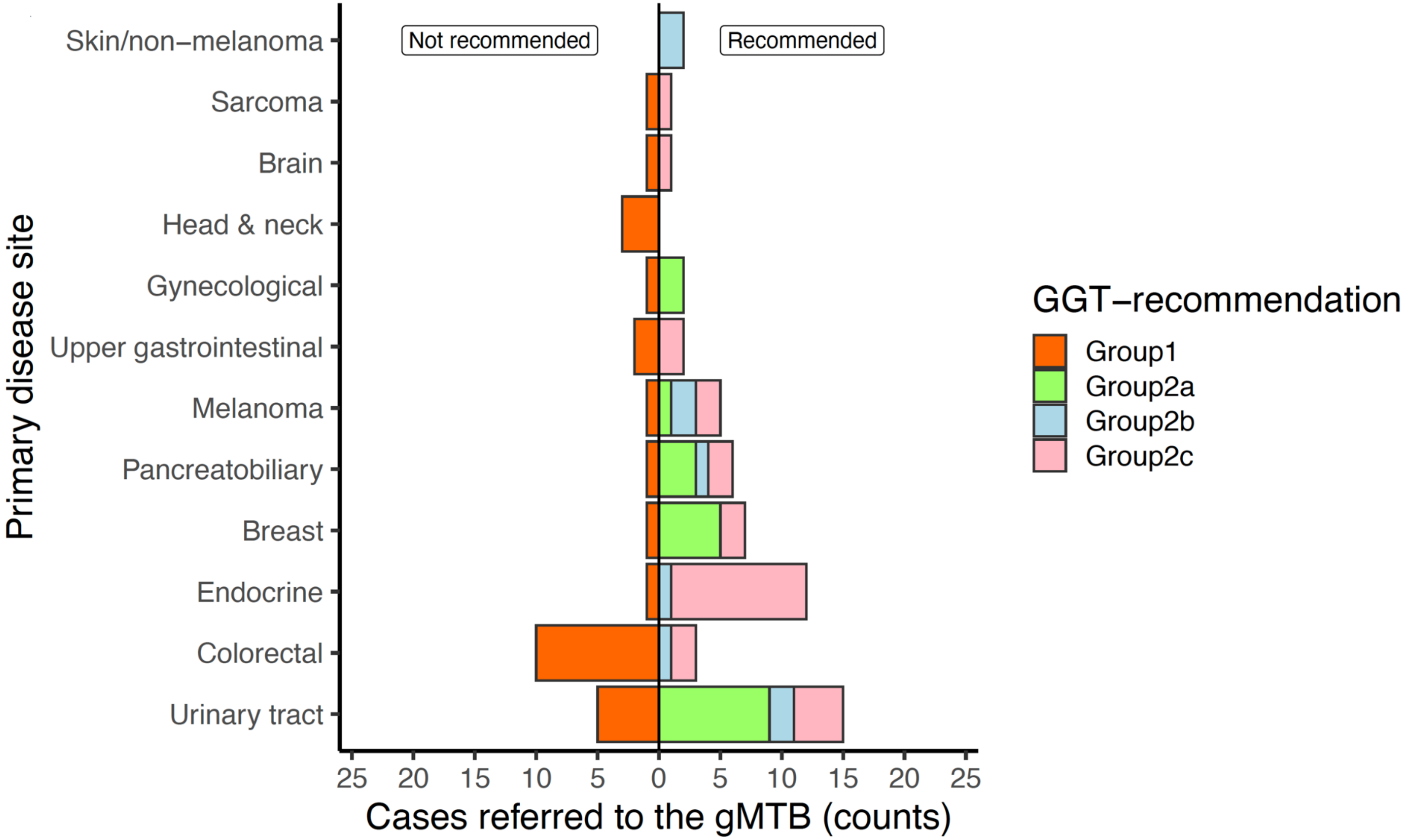

Supplementary Fig. 2 Distribution of 83 cases referred to the gMTB by cancer site across all the groups: Group 1 – GGT Not recommended (red); Group 2 – GGT Recommended: 2A (green), 2B (light blue) and 2C (pink). gMTB, germline molecular tumor board; GGT, germline genetic testing

Supplementary Table 2. Description of OCTANE cases not recommended for germline testing by the gMTB (n = 27) – Group 1

Age at diagnosis	Sex	Cancer type	Tumor Genetic Variant (TGV, n = 40)				Reasons to not pursue germline confirmation (GGT)	
			**Gene**	**HGVS nomenclature**	**VAF %**	**Tier**	**As per ESMO and similar guidelines "tumor-only criteria"**	**As per personal/family history "germline criteria"**
71	Female	Colorectal cancer	*APC*	NM_000038.6:c.4271del p.(Pro1424GlnfsTer49)	76%	II	Diagnosis after 30 years (low GCR)	No personal or family history of related phenotypes (i.e., >10 adenomas; juvenile polyps)
			SMAD4	NM_005359.6:c.1561del p.(Thr521HisfsTer16)	70%	II		
82	Male	Bladder cancer	*ATM*	NM_000051.4:c.6600del p.(Val2201TyrfsTer34)	21%	II	*ATM* and *CDKN2A* excluded as per ESMO 2019	PHx: Dermatofibrosarcoma (77); FDR: Lung (d.70); Colon (d.60's)
			CDKN2A	NM_000077.5:c.57del p.(Ala20ArgfsTer6)	46%	II	*CDKN2A,* Diagnosis after 30 years (low GCR) and Off-tumor context (low GCR)	
52	Male	Bladder cancer	*TSC1*	NM_000368.5:c.2356C>T p.(Arg786Ter)	34%	II	Overall low GCR	No relevant personal or family history
34	Male	Nasopharyngeal carcinoma	*CHEK2*	NM_007194.4:c.1556G>T p.(Arg519Leu)	63%	ND	It is a VUS	No relevant personal or family history
52	Female	Gastric adenocarcinoma	*SMAD4*	NM_005359.6:c.1610A>G p.(Asp537Gly)	67%	III	*SMAD4* is a VUS	No personal or family history of related phenotypes (i.e., juvenile polyps)
61	Female	Cholangio-carcinoma	*MUTYH*	NC_000001.11(NM_001048174.2):c.850-2A>G p.?	54%	III	Conflicting interpretation in ClinVar and not bi-allelic (not relevant for the patient)	No relevant personal or family history
72	Male	Colorectal cancer	*APC*	NM_000038.6:c.643C>T p.(Gln215Ter)	23%	II	Diagnosis after 30 years (low GCR)	PHx: Prostate - no high-risk group (68); 5-6 polyps - no high-grade dysplasia; FDR: Liver (d.90); SDR: Unk Primary (d.80's), Breast (d.80's)
68	Male	Sinus adenocarcinoma	*CDKN2A*	NM_000077.5:c.238C>T p.(Arg80Ter)	33%	II	Diagnosis after 30 years (low GCR) and Off-tumor context (low GCR)	No relevant personal or family history of related phenotypes (i.e, melanoma, pancreatic cancer)
			CDKN2A	NM_000077.5:c.329G>A p.(Trp110Ter)	37%	II		
58	Female	Clear cell renal cell carcinoma	*VHL*	NM_000551.4:c.259_296del p.(Val87AsnfsTer32)	46%	I	On-tumor context (i.e., Kidney with low GCR)	No relevant personal or family history (i.e., diagnosis before 45 years, bilateral presentation, etc)

73	Female	Urothelial cancer	*BAP1*	NM_004656.4:c.505C>T p.(His169Tyr)	92%	II	It is a VUS	FDR: Lung (57)
60	Female	Colorectal cancer	*APC*	NM_000038.6:c.4666dup p.(Thr1556AsnfsTer3)	23%	II	Diagnosis after 30 years (low GCR)	No personal or family history of related phenotypes (i.e., >10 adenomas)
76	Male	Glioblastoma	*RB1*	NC_000013.11(NM_000321.3):c.253_264+6del p.?	42%	III	Diagnosis after 30 years (low GCR)	No relevant personal or family history
			PTEN	NM_000314.8:c.380G>A p.(Gly127Glu)	8%	II		
74	Female	Colorectal cancer	*APC*	NM_000038.6:c.3927_3931del p.(Glu1309AspfsTer4)	36%	II	Diagnosis after 30 years (low GCR)	No personal or family history of related phenotypes (i.e., >10 adenomas)
39	Male	Melanoma	*CDKN2A*	NM_000077.5:c.242C>T p.(Pro81Leu)	46%	II	*CDKN2A* excluded as per ESMO 2019 and Diagnosis after 30 years (low GCR)	FDR: Lymphoma (Mother); FDR: Leukemia (Father)
			MUTYH	NM_001048174.2:c.1103G>A p.(Gly368Asp)	45%	II	*MUTYH* not bi-allelic (not relevant for the patient)	
62	Female	Colorectal cancer	*APC*	NM_000038.6:c.4364del p.(Asn1455IlefsTer18)	20%	II	Diagnosis after 30 years (low GCR)	No personal or family history of related phenotypes (i.e., >10 adenomas)
			APC	NM_000038.6:c.2626C>T p.(Arg876Ter)	26%	II		
67	Male	Salivary gland adenocarcinoma	*NF1*	NM_001042492.3:c.952_953del p.(Glu318LysfsTer11)	30%	II	*NF1*, as per ESMO 2019 is Off-tumor context (low GCR) and Diagnosis after 30 years (low GCR)	No personal or family history of related phenotypes (i.e., multiple café-au-lait macules, skinfold freckling, cutaneous neurofibromas, etc)
42	Female	Colorectal cancer	*APC*	NM_000038.6:c.637C>T p.(Arg213Ter)	26%	II	Diagnosis after 30 years (low GCR)	FDR: Lung; SDR: Lung (#2), Prostate (#2)
			APC	NM_000038.6:c.4463del p.(Leu1488TyrfsTer19)	27%	II		No personal or family history of related phenotypes (i.e., >10 adenomas)
52	Female	Colorectal cancer	*APC*	NM_000038.6:c.637C>T p.(Arg213Ter)	67%	II	Diagnosis after 30 years (low GCR)	No personal or family history of related phenotypes (i.e., >10 adenomas)
			ATM	NM_000051.4:c.8495G>A p.(Arg2832His)	76%	III	*ATM* is a VUS	
73	Male	Colorectal cancer	*EPCAM*	NM_002354.3:c.316A>T p.(Lys106Ter)	49%	II	Only large deletions of *EPCAM* (3'-UTR, including exon 8 and exon 9) are relevant for Lynch Syndrome	No relevant personal or family history
65	Male	Colorectal cancer	*APC*	NM_000038.6:c.1779G>A p.(Trp593Ter)	24%	II	Diagnosis after 30 years (low GCR)	No personal or family history of related phenotypes (i.e., >10 adenomas)
			APC	NM_000038.6:c.4128T>A p.(Tyr1376Ter)	31%	II		

50	Female	Colorectal cancer	*APC*	NM_000038.6:c.4348C>T p.(Arg1450Ter)	23%	II	Diagnosis after 30 years (low GCR)	No personal or family history of related phenotypes (i.e., >10 adenomas)
			MAX	NM_002382.5:c.179G>A p.(Arg60Gln)	20%	III	*MAX* is a VUS	
68	Male	Thyroid cancer	*MEN1*	NM_001370259.2:c.358_360del p.(Lys120del)	86%	II	Overall low GCR	No relevant personal or family history
55	Female	Endometrial cancer	*PTEN*	NM_000314.8:c.179del p.(Lys60SerfsTer39)	31%	II	Diagnosis after 30 years (low GCR)	FDR: Leukemia; SDR: Lung
			PTEN	NC_000010.11(NM_000314.8):c.492+1del p.?	34%	II		No relevant personal or family history
64	Male	Small bowel adenocarcinoma	*SMAD4*	NM_005359.6:c.1156G>A p.(Gly386Ser)	57%	III	Diagnosis after 30 years (low GCR)	PHx: Pituitary microadenoma. FHx: FDR: Breast, Unk Primary. No personal or family history of related phenotypes (i.e., juvenile polyps)
64	Male	Leiomyosarcoma	*MUTYH*	NM_001048174.2:c.1354G>T p.(Glu452Ter)	64%	II	*MUTYH* not bi-allelic (not relevant for the patient)	No relevant personal or family history
			RB1	NC_000013.11(NM_000321.3):c.138-21_140del p.?	79%	III	Diagnosis after 30 years (low GCR)	
58	Male	Clear cell renal cell carcinoma	*VHL*	NC_000003.12(NM_000551.4):c.463+1del p.?	18%	I	On-tumor context (i.e., Kidney with low GCR)	No relevant personal or family history
			PTEN	NM_000314.8:c.481A>T p.(Arg161Ter)	33%	II	Diagnosis after 30 years (low GCR)	
59	Female	Breast cancer	*NF1*	NC_000017.11(NM_001042492.3):c.7189+1G>C p.?	57%	II	On-tumor context (i.e., Breast with low GCR)	No personal or family history of related phenotypes (i.e., multiple café-au-lait macules, skinfold freckling, cutaneous neurofibromas, etc)

OCTANE, the Ontario-wide Cancer Targeted Nucleic Acid Evaluation (OCTANE) clinical trial [11]; gMTB, germline molecular tumor board; HGVS, Human Genome Variation Society; VAF, variant allele fraction; VUS, variant of uncertain significance; ESMO, European Society for Medical Oncology; GCR, germline conversion rate; Tumor-only criteria, based on recommendation for germline confirmation of TGVs as per tumor-only guidelines [1-10]; Germline criteria, based on the Cancer Care Ontario Hereditary Cancer Testing Eligibility Criteria [14]; 3'-UTR, three prime untranslated region; PHx, personal history of cancer; FHx, family history of cancer, FDR, first degree relative; SDR, second degree relative; Unk, unknown. TGVs in grey, corresponds to those considered *not germline relevant*.

Supplementary Table 3. Description of OCTANE cases recommended for germline testing by the gMTB based only on “germline criteria” (n = 20) – Group 2a

Age at diagnosis	Sex	Cancer type	Tumor Genetic Variant (TGV, n = 26)				Germline Genetic Testing (GGT)			Germline variant		Tumor - Germline Variants Correlation		
			**Gene**	**HGVS nomenclature**	**VAF %**	**Tier**	**Prior to tumor testing**	**Test type**	**Result**	**Gene**	**HGVS nomenclature**	**Yes**	**No**	**Unknown**
“Germline criteria” supporting the suspicion of a HCS:														
**Personal history of metastatic prostate cancer (n = 5)**														
70	Male	Prostate cancer	*RB1*	NM_000321.3:c.1959dup p.(Val654SerfsTer14)	26%	II	Yes	Panel	Negative					*RB1*
74	Male	Prostate cancer	*CDKN1B*	NM_004064.5:c.182del p.(Asn61IlefsTer10)	66%	III	Yes	Panel	Negative					*CDKN1B*
78	Male	Prostate cancer	*APC*	NM_000038.6:c.5833del p.(Ala1945GlnfsTer25)	24%	II	Yes	Panel	VUS	*BRCA2*	NM_000059.4:c.9863C>T p.(Thr3288Ile)		*BRCA2*<br>*CHEK2*	*APC*
			APC	NM_000038.6:c.4666dup p.(Thr1556AsnfsTer3)	18%	II				*CHEK2*	NM_007194.4:c.288A>T p.(Leu96Phe)			
69	Male	Prostate cancer	*FANCA*	NM_000135.4:c.2738A>C p.(His913Pro)	93%	II	No	Panel	Negative					*FANCA*
58	Male	Prostate cancer	*CDKN1B*	NM_004064.5:c.115G>T p.(Glu39Ter)	30%	III	No	Recommended	Not Performed					*CDKN1B*
**Personal history of pancreatic cancer (n = 3)**														
59	Male	Pancreatic cancer	*CDKN2A*<br>*NBN*<br>*SMAD4*	NC_000009.12(NM_000077.5):c.151-2A>G p.?<br>NM_002485.5:c.657_661del p.(Lys219AsnfsTer16)<br>NM_005359.6:c.1139G>A p.(Arg380Lys)	27%<br>55%<br>9%	II<br>III<br>II	Unknown	Recommended	Not Performed					*CDKN2A*<br>*NBN*<br>*SMAD4*
35	Male	Pancreatic cancer	*CDKN2A*	NM_000077.5:c.235dup p.(Thr79AsnfsTer41)	47%	II	Yes	Panel	Negative				*CDKN2A*	
74	Female	Pancreatic cancer	*SMAD4*	NM_005359.6:c.1333C>T p.(Arg445Ter)	32%	II	Yes	Panel	VUS	*PTCH1*	NM_000264.5:c.172G>A p.(Ala58Thr)		*PTCH1*<br>*SMAD4*	
**Personal history of renal cell carcinoma before 45 years (n = 2); family history of prostate cancer (n = 1); chromophobe component (n = 1)**														
39	Female	Clear cell renal cell carcinoma	*VHL*	NM_000551.4:c.506T>C p.(Leu169Pro)	27%	I	No	Panel	VUS	*SDHD*	NM_003002.4:c.335C>T p.(Thr112Ile)		*SDHD*<br>*VHL*	
44	Male		*PTEN*	NC_000010.11(NM_000314.8):c.210-1G>T p.?	5%	II	No	Recommended	Not Performed					*PTEN*<br>*VHL*

		Clear cell renal cell carcinoma	*VHL*	NM_000551.4:c.353T>C p.(Leu118Pro)	7%	I								
51	Male	Renal cell carcinoma	*VHL*	NM_000551.4:c.444del p.(Phe148LeufsTer11)	34%	I	Yes	Panel	Negative					*VHL*
71	Male	Renal cell carcinoma	*PTEN*	NM_000314.8:c.758T>A p.(Ile253Asn)	71%	II	No	Recommended	Not Performed					*PTEN*
**Personal history of metastatic breast cancer: triple negative before 60 years (n = 1); diagnosis before 45 years (n = 2); medical judgment (n = 2)**														
50	Female	Breast cancer	*MSH2*	NM_000251.3:c.1774A>G p.(Met592Val)	59%	III	Yes	Panel	VUS	*MSH2*	NM_000251.3:c.1774A>G p.(Met592Val)	*MSH2*		
41	Female	Breast cancer	*CDH1*	NC_000016.10(NM_004360.5):c.49-2del p.?	42%	II	Yes	Panel	VUS	*BRIP1*	NM_032043.3:c.1735C>T p.(Arg579Cys)		*CDH1*<br>*BRIP1*<br>*PMS2/PMS2CL*<br>*RAD51D*	
										PMS2/PMS2CL	NM_000535.7:c.1145-?_2589+?dup (p.?)			
										RAD51D	NM_002878.4:c.573G>A p.(Gln191=)			
57	Female	Breast cancer	*CDH1*	NM_004360.5:c.2095C>T p.(Gln699Ter)	62%	II	No	Panel	Negative				*CDH1*	
40	Female	Breast cancer	*PTEN*	NM_000314.8:c.45A>C p.(Arg15Ser)	36%	II	Yes	Panel	Negative				*PTEN*	
70	Female	Breast cancer	*CDH1*	NM_004360.5:c.94_96del p.(Phe32del)	45%	II	Yes	BRCA1-2 AJ mutations	Negative					*CDH1*
**Others: Personal history of endometrial cancer with relevant family history (n = 2) and melanoma in a case with multiple primaries (n = 1)**														
38	Female	Endometrial cancer	*PTEN*	NC_000010.11(NM_000314.8):c.210-2A>T p.?	21%	II	Yes	Panel	Negative				*PTEN*	
			PTEN	NM_000314.8:c.775del p.(His259ThrfsTer7)	20%	II								
67	Female	Endometrial cancer	*PTEN*	NM_000314.8:c.69_74del p.(Asp24_Leu25del)	38%	II	Yes	Panel	Negative				*PTEN*	
88	Male	Melanoma	*PRKAR1A*	NM_002734.5:c.682C>T p.(Arg228Ter)	47%	III	No	Recommended	Not Performed					*PRKAR1A*<br>*RB1*
			RB1	NC_000013.11(NM_000321.3):c.1050-1G>C p.?	42%	III								

OCTANE, the Ontario-wide Cancer Targeted Nucleic Acid Evaluation (OCTANE) clinical trial [11]; gMTB, germline molecular tumor board; HCS, hereditary cancer syndrome; HGVS, Human Genome Variation Society; VAF, variant allele fraction; AJ, Ashkenazi Jewish; Germline criteria, patients met the Cancer Care Ontario Hereditary Cancer Testing Eligibility Criteria [14]. TGVs in grey, corresponds to those considered *not germline relevant*. Regardless of the TGV, all these patients received a recommendation for GGT due to the clinical suspicion of a HCS (i.e., all met "germline criteria" for GGT) [14].

Supplementary Table 4. Description of OCTANE cases with tumor variants recommended for germline confirmation by the gMTB based solely on "tumor-only criteria" (n = 9) – Group 2b

Age at diagnosis	Sex	Cancer type	Tumor Genetic Variant (TGV, n = 26)				Germline Genetic Testing (GGT)			Germline variant		Tumor - Germline Variants Correlation		
			**Gene**	**HGVS nomenclature**	**VAF %**	**Tier**	**Prior to tumor testing**	**Test type**	**Result**	**Gene**	**HGVS nomenclature**	**Yes**	**No**	**Unknown**
66	Male	Bladder cancer	*BRCA1*	NM_007294.4:c.280C>T p.(Gln94Ter)	37%	II	No	Recommended	Not Performed					*BRCA1*
60	Male	Cholangio-carcinoma	*BAP1*<br>*NBN*	NC_000003.12(NM_004656.4):c.375+2T>A p.?<br>NM_002485.5:c.511A>G p.(Ile171Val)	26%<br>42%	II<br>III	No	Recommended	Not Performed					*BAP1*<br>*NBN*
50	Male	Porocarcinoma	*BRCA2*<br>*HOXB13*<br>*RB1*	NM_000059.4:c.5142T>A p.(Tyr1714Ter)<br>NM_006361.6:c.251G>A p.(Gly84Glu)<br>NM_000321.3:c.1649T>G p.(Leu550Ter)	14%<br>53%<br>25%	II<br>III<br>III	No	Recommended	Not Performed					*BRCA2*<br>*HOXB13*<br>*RB1*
48	Male	Basal cell carcinoma	*PTCH1*	NM_000264.5:c.2050G>T p.(Glu684Ter)	82%	II	No	Recommended	Not Performed					*PTCH1*
**29**	**Female**	**Thyroid cancer**	***ATM***	**NM_000051.4:c.7495G>T p.(Glu2499Ter)**	**48%**	**II**	**No**	**Specific mutation**	**Positive**	***ATM***	**NM_000051.4:c.7495G>T p.(Glu2499Ter)**	***ATM***		
82	Male	Melanoma	*CDKN2A*<br>*NF2*	NC_000009.12(NM_000077.5):c.151-1G>A p.?<br>NM_000268.4:c.168_169delinsGT p.(Arg57Ter)	24%<br>26%	II<br>II	No	Recommended	Not Performed					*CDKN2A*<br>*NF2*
59	Male	Colorectal cancer	*APC*<br>*APC*<br>*AXIN2*	NM_000038.6:c.2991del p.(Tyr997Ter)<br>NM_000038.6:c.4348C>T p.(Arg1450Ter)<br>NM_004655.4:c.38_39dup p.(Ser14ProfsTer63)	32%<br>31%<br>40%	II<br>II<br>III	No	Recommended	Not Performed					*APC*<br>*AXIN2*
69	Male	Renal cell carcinoma	*VHL*<br>*ATM*<br>*BAP1*<br>*CDKN2A*<br>*PTCH1*	NM_000551.4:c.221_222delinsAT p.(Val74Asp)<br>NM_000051.4:c.5674G>T p.(Glu1892Ter)<br>NM_004656.4:c.279dup p.(His94SerfsTer32)<br>NM_000077.5:c.68del p.(Gly23ValfsTer3)<br>NM_000264.5:c.3921del p.(Arg1308GlufsTer64)	32%<br>23%<br>33%<br>24%<br>26%	I<br>II<br>II<br>II<br>II	No	Panel	Negative				*BAP1*<br>*VHL*	*ATM*<br>*CDKN2A*<br>*PTCH1*
33	Female	Melanoma	*CDKN2A*	NM_000077.5:c.205G>T p.(Glu69Ter)	22%	II	No	Recommended	Not Performed					*CDKN2A*

OCTANE, the Ontario-wide Cancer Targeted Nucleic Acid Evaluation (OCTANE) clinical trial [11]; gMTB, germline molecular tumor board; Tumor-only criteria, the TGV met the recommendation for germline confirmation as per tumor-only guidelines [1-10]; HGVS, Human Genome Variation Society; VAF, variant allele fraction. TGVs in grey, corresponds to those considered *not germline relevant*. TGVs in black, corresponds to those that were recommended for germline confirmation (i.e., *germline relevant*). TGVs in bold, corresponds to those that are interpreted on the germline as P/LP and were confirmed as *true germline variants.*

Supplementary Table 5. Description of OCTANE cases recommended for germline confirmation by the gMTB based on “germline criteria” and “tumor-only criteria” (n = 27) – Group 2c

Age at diagnosis	Sex	Cancer type	Tumor Genetic Variant (TGV, n = 26)				Germline Genetic Testing (GGT)			Germline variant		Tumor - Germline Variants Correlation		
			Gene	HGVS nomenclature	VAF %	Tier	Prior to tumor testing	Test type	Result	Gene	HGVS nomenclature	Yes	No	Unknown
**50**	**Female**	**Adrenal cortical carcinoma**	***FLCN***	**NM_144997.7:c.694C>T p.(Gln232Ter)**	**83%**	**III**	**Yes**	**Panel**	**Positive**	***FLCN***	**NM_144997.7:c.694C>T p.(Gln232Ter)**	***FLCN***		
69	Male	Prostate cancer	*MSH2*	NM_000251.3:c.2599G>T p.(Glu867Ter)	39%	I	Yes	*BRCA1-2*<br>Specific mutation	Negative<br>*MSH2* not performed					*MSH2*
61	Female	Pancreatic cancer	*HOXB13*	NM_006361.6:c.251G>A p.(Gly84Glu)	23%	II	Yes	Panel<br>Specific mutation	Negative<br>*HOXB13* not performed					*HOXB13*
48	Female	Thyroid cancer	*RET*	NM_020975.6:c.2753T>C p.(Met918Thr)	41%	I	Unknown	Recommended	Not Performed					*RET*
59	Male	Prostate cancer	*RNF43*	NC_000017.11(NM_017763.6):c.450+2T>C p.?	33%	II	Yes	Panel<br>Specific mutation	Negative				*RNF43*	
54	Female	Pancreatic cancer	*CDKN2A*<br>*MUTYH*	NM_000077.5:c.238C>T p.(Arg80Ter)<br>NM_001048174.2:c.1103G>A p.(Gly368Asp)	36%<br>52%	II<br>II	Yes	Panel<br>Extended panel<br>Specific mutation	VUS<br>*CDKN2A* not performed<br>*MUTYH* not performed	*ATM*	NM_000051.4:c.6335G>A p.(Cys2112Tyr)		*ATM*	*CDKN2A*<br>*MUTYH*
**57**	**Female**	**Thymoma**	***RET***	**NM_020975.6:c.2410G>A p.(Val804Met)**	**49%**	**II**	**Yes**	**Panel**	**Positive**	***RET***	**NM_020975.6:c.2410G>A p.(Val804Met)**	***RET***		
47	Female	Pheochromocytoma	*MEN1*<br>*NF1*	NC_000011.10(NM_001370259.2):c.783+1G>C p.?<br>NM_001042492.3:c.1466A>G p.(Tyr489Cys)	59%<br>53%	II<br>II	Yes	Panel	Negative				*MEN1*<br>*NF1*	
**68**	**Male**	**Prostate cancer**	***CHEK2***	**NM_007194.4:c.470T>C p.(Ile157Thr)**	**47%**	**I**	**Yes**	**Panel**	**Positive**	***CHEK2***	**NM_007194.4:c.470T>C p.(Ile157Thr)**	***CHEK2***	*TSC2*	

			TSC2	NM_000548.5:c.3203_3216del p.(Thr1068SerfsTer95)	30%	II		Specific mutation						
23	Male	Gastric diffuse-type	*CDH1*<br>*TP53*	NC_000016.10(NM_004360.5):c.1009-4_1012del p.?<br>NM_000546.6:c.916C>T p.(Arg306Ter)	27%<br>19%	II<br>II	Yes	Panel	VUS	*APC*<br>*GALNT12*	NM_000038.6:c.1455G>A p.(Met485Ile)<br>NM_024642.5:c.329G>A p.(Arg110His)		*CDH1*<br>*TP53*<br>*APC*<br>*GALNT12*	
**32**	**Female**	**Breast cancer**	***BRCA1***	**NM_007294.4:c.5106del p.(Lys1702AsnfsTer4)**	**60%**	**I**	**Yes**	**Panel**	**Positive**	***BRCA1***	**NM_007294.4:c.5106del p.(Lys1702AsnfsTer4)**	***BRCA1***		
28	Female	Soft tissue sarcoma	*CDH1*<br>*TERT*<br>*TP53*	NM_004360.5:c.2195G>A p.(Arg732Gln)<br>NC_000005.10:g.1295135G>A<br>NM_000546.6:c.818G>A p.(Arg273His)	37%<br>58%<br>67%	III<br>III<br>II	No	Panel<br>Specific mutation	VUS	*BRCA1*	NM_007294.4:c.5068A>C p.(Lys1690Gln)		*TP53*<br>*CDH1*<br>*BRCA1*	*TERT*
59	Female	Choroidal melanoma	*BAP1*<br>*MUTYH*	NM_004656.4:c.358A>T p.(Lys120Ter)<br>NM_001048174.2:c.452A>G p.(Tyr151Cys)	78%<br>12%	I<br>II	Yes	Panel	Negative				*BAP1*	*MUTYH*
47	Male	Paraganglioma	*SDHB*	NM_003000.3:c.260T>C p.(Leu87Ser)	57%	II	Yes	Panel	Negative				*SDHB*	
**55**	**Male**	**Colorectal cancer**	***CHEK2***	**NM_007194.4:c.1427C>T p.(Thr476Met)**	**36%**	**II**	**Yes**	**Panel**	**Positive**	***CHEK2***	**NM_007194.4:c.1427C>T p.(Thr476Met)**	***CHEK2***		
32	Female	Thyroid cancer	*RET*	NM_020975.6:c.1901G>A p.(Cys634Tyr)	33%	I	Yes	Panel	Negative				*RET*	
56	Female	Clear cell renal cell carcinoma	*VHL*<br>*TERT*	NM_000551.4:c.521del p.(Asn174IlefsTer28)<br>NC_000005.10:g.1295113G>A	47%<br>32%	I<br>III	No	Panel	VUS	*TSC2*	NM_000548.5:c.1678G>A p.(Val560Met)		*VHL*<br>*TSC2*	*TERT*
**25**	**Male**	**Colorectal cancer**	***MLH1***	**NC_000003.12(NM_000249.4):c.1667+1G>T p.?**	**59%**	**II**	**Yes**	**Panel**	**Positive**	***MLH1***	**NC_000003.12(NM_000249.4):c.1667+1G>T p.?**	***MLH1***		
69	Male	Thyroid cancer	*RET*	NM_020975.6:c.2753T>C p.(Met918Thr)	48%	I	Yes	Panel	Negative				*RET*	
58	Female	Melanoma	*MUTYH*<br>*NF1*	NM_001048174.2:c.452A>G p.(Tyr151Cys)<br>NM_001042492.3:c.4084C>T p.(Arg1362Ter)	66%<br>44%	III<br>II	Yes	Panel<br>Specific mutation	Negative<br>*SDHAF2* not performed					*SDHAF2*<br>*SDHD*<br>*MUTYH*<br>*NF1*<br>*RB1*

			RB1	NM_000321.3:c.1915C>T p.(Gln639Ter)	23%	III			*SDHD* not performed					
			SDHAF2	NM_017841.4:c.444_447del p.(Asn148LysfsTer34)	50%	II								
			SDHD	NM_003002.4:c.304C>T p.(His102Tyr)	45%	III								
37	Female	Gastric diffuse-type	*RNF43*	NM_017763.6:c.1247G>A p.(Trp416Ter)	50%	II	Yes	Panel<br>Specific mutation	Negative<br>*RNF43* not performed					*RNF43*
**33**	**Female**	**Diffuse pediatric high-grade glioma**	***MSH2***	**NM_000251.3:c.425C>G p.(Ser142Ter)**	**76%**	**II**	**Yes**	**Panel**	**Positive**	***MSH2***	**NM_000251.3:c.425C>G p.(Ser142Ter)**	***MSH2***	*PTEN*<br>*NF1*	
			NF1	NM_001042492.3:c.5246_5247del p.(Lys1749ArgfsTer7)	24%	III								
			NF1	NM_001042492.3:c.5907_5910del p.(Arg1970LeufsTer8)	22%	III								
			PTEN	NM_000314.8:c.1048dup p.(Thr350AsnfsTer11)	29%	II								
69	Male	Thyroid cancer	*RET*	NM_020975.6:c.1900T>C p.(Cys634Arg)	49%	I	No	Recommended	Not Performed					*RET*
37	Male	Thyroid cancer	*RET*	NM_020975.6:c.2753T>C p.(Met918Thr)	35%	I	Yes	Panel	Negative				*RET*	
**35**	**Female**	**Breast cancer**	***BRCA2***	**NM_000059.4:c.5576_5579del p.(Ile1859LysfsTer3)**	**83%**	**I**	**Yes**	**Panel**	**Positive**	***BRCA2***	**NM_000059.4:c.5576_5579del p.(Ile1859LysfsTer3)**	***BRCA2***		
37	Female	Thyroid cancer	*RET*	NM_020975.6:c.1888T>C p.(Cys630Arg)	41%	I	Yes	Panel	Negative				*RET*	
43	Female	Thyroid cancer	*RET*	NM_020975.6:c.1902C>G p.(Cys634Trp)	47%	I	Yes	Panel	Negative				*RET*	

OCTANE, the Ontario-wide Cancer Targeted Nucleic Acid Evaluation (OCTANE) clinical trial [11]; gMTB, molecular tumor board; Germline criteria, patients met the Cancer Care Ontario Hereditary Cancer Testing Eligibility Criteria [14]; Tumor-only criteria, the TGV met the recommendation for germline confirmation as per tumor-only guidelines [1-10]; HGVS, Human Genome Variation Society; VAF, variant allele fraction; VUS, variant of uncertain significance. TGVs in grey, corresponds to those considered *not germline relevant*. TGVs in black, corresponds to those that were recommended for germline confirmation (i.e., *germline relevant*). TGVs in bold, corresponds to those that are interpreted on the germline as P/LP and were confirmed as *true germline variants.*

Supplementary Table 6. Description of 127 TGVs in 37 genes, reviewed by the gMTB from 83 cases with tumor genomic profiling results from OCTANE clinical trial

Genes (n = 37)	TGV (n = 127)	Results of TGVs assessment per Group								Total TGV recommended for GGT (%)	
		GGT not indicated - Group 1		GGT indicated - Group 2							
		Group 1 - Did not meet any criteria 'germline and tumor-only' (27 cases; 40 TGVs)		Group 2a - Only met 'germline criteria' (20 cases; 26 TGVs)		Group 2b - Only met 'tumor-only criteria' (9 cases; 19 TGVs)		Group 2c - Met both criteria 'germline and tumor-only' (27 cases; 42 TGVs)			
Clinical Actionability by ESMO		Germline Relevant		Germline Relevant		Germline Relevant		Germline Relevant			
**5 Most-actionable (MA)**	**TGV**	**Yes**	**No**	**Yes**	**No**	**Yes**	**No**	**Yes**	**No**	**Yes**	**No**
BRCA1	2	0	0	0	0	1	0	1	0	2 (100%)	0
BRCA2	2	0	0	0	0	1	0	1	0	2 (100%)	0
MLH1	1	0	0	0	0	0	0	1	0	1 (100%)	0
MSH2	3	0	0	0	1	0	0	2	0	2 (66.7%)	1 (33.3%)
RET	8	0	0	0	0	0	0	8	0	8 (100%)	0
**Total**	**16 (12.6%)**	**0**	**0**	**0**	**1**	**2**	**0**	**13**	**0**	**15 (93.8%)**	**1 (6.2%)**
**15 High-actionability (HA)**	**TGV**	**Yes**	**No**	**Yes**	**No**	**Yes**	**No**	**Yes**	**No**	**Yes**	**No**
APC	16	0	12	0	2	0	2	0	0	0	16 (100%)
MAX	1	0	1	0	0	0	0	0	0	0	1 (100%)
MEN1	2	0	1	0	0	0	0	1	0	1 (50.0%)	1 (50.0%)
MUTYH	6	0	3	0	0	0	0	1	2	1 (16.7%)[a]	5 (83.3%)[a]
NF2	1	0	0	0	0	0	1	0	0	0	1 (100%)
PTEN	11	0	4	0	6	0	0	0	1	0	11 (100%)
RB1	6	0	2	0	2	0	1	0	1	0	6 (100%)
SDHAF2	1	0	0	0	0	0	0	1	0	1 (100%)	0
SDHB	1	0	0	0	0	0	0	1	0	1 (100%)	0

SDHD	1	0	0	0	0	0	0	1	0	1 (100%)	0
SMAD4	5	0	3	0	2	0	0	0	0	0	5 (100%)
TP53	2	0	0	0	0	0	0	2	0	2 (100%)	0
TSC1	1	0	1	0	0	0	0	0	0	0	1 (100%)
TSC2	1	0	0	0	0	0	0	1	0	1 (100%)	0
VHL	7	0	2	0	3	0	1	1	0	1 (14.3%)	6 (85.7%)
**Total**	**62 (48.8%)**	**0**	**29**	**0**	**15**	**0**	**5**	**9**	**4**	**9 (14.5%)**	**53 (85.5%)**
**9 Standard-actionability (SA)**	**TGV**	**Yes**	**No**	**Yes**	**No**	**Yes**	**No**	**Yes**	**No**	**Yes**	**No**
ATM	4	0	2	0	0	1	1	0	0	1 (25.0%)	3 (75.0%)
BAP1	4	0	1	0	0	2	0	1	0	3 (75.0%)	1 (25.0%)
CDH1	5	0	0	0	3	0	0	1	1	1 (20.0%)	4 (80.0%)
CDKN2A	10	0	4	0	2	2	1	1	0	3 (30.0%)	7 (70.0%)
CHEK2	3	0	1	0	0	0	0	2	0	2 (66.7%)[a]	1 (33.3%)
FLCN	1	0	0	0	0	0	0	1	0	1 (100%)	0
NF1	6	0	2	0	0	0	0	3	1	3 (50.0%)	3 (50.0%)
PTCH1	2	0	0	0	0	1	1	0	0	1 (50.0%)	1 (50.0%)
TERT	2	0	0	0	0	0	0	0	2	0	2 (100%)
**Total**	**37 (29.1%)**	**0**	**10**	**0**	**5**	**6**	**3**	**9**	**4**	**15 (40.5%)**	**22 (59.5%)**
**8 Not included in ESMO**	**TGV**	**Yes**	**No**	**Yes**	**No**	**Yes**	**No**	**Yes**	**No**	**Yes**	**No**
AXIN2	1	0	0	0	0	1	0	0	0	1 (100%)	0
CDKN1B	2	0	0	0	2	0	0	0	0	0	2 (100%)
EPCAM	1	0	1	0	0	0	0	0	0	0	1 (100%)
FANCA	1	0	0	0	1	0	0	0	0	0	1 (100%)
HOXB13	2	0	0	0	0	1	0	1	0	2 (100%)[a]	0
NBN	2	0	0	0	1	0	1	0	0	0	2 (100%)

PRKAR1A	1	0	0	0	1	0	0	0	0	0	1 (100%)
RNF43	2	0	0	0	0	0	0	2	0	2 (100%)	0
**Total**	**12 (9.5%)**	**0**	**1**	**0**	**5**	**2**	**1**	**3**	**0**	**5 (41.7%)**	**7 (58.3%)**
**TOTAL**	**127 (100%)**	**0**	**40**	**0**	**26**	**10**	**9**	**34**	**8**	**44 (34.6%)**	**83 (65.4%)**

TGV, tumor genetic variants; gMTB, molecular tumor board; OCTANE, the Ontario-wide Cancer Targeted Nucleic Acid Evaluation (OCTANE) clinical trial [11]; GGT, germline genetic testing/confirmation; Germline criteria, based on the Cancer Care Ontario Hereditary Cancer Testing Eligibility Criteria [14]; Tumor-only criteria, based on the recommendation for germline confirmation of TGVs as per tumor-only guidelines [1-10]; ESMO, The European Society of Medical Oncology (ESMO) Precision Medicine Working Group Germline Subgroup [1, 4].

a: Includes known founder mutations [1, 3, 14-20] detected in *CHEK2* (I157T, n = 1), *HOXB13* (G84E, n = 2) and *MUTYH* (G368D, n = 2 and Y151C, n = 2). All these founder mutations were the only mutations reported in these genes for 7/83 cases. Founder mutations in *CHEK2* and *HOXB13* genes were recommended for germline confirmation. Only one case with relevant personal and family history for MAP (*MUTYH*-Associated Polyposis) syndrome and a founder mutation in *MUTYH* (G368D) was recommended for germline follow-up. Following ESMO tumor-only guidelines [1, 4], germline confirmation was not directly recommended for any other case with a *MUTYH* mutation (including three cases with founder mutations), since none of these cases had two pathogenic variants detected in *MUTYH* (Supplementary Table 7).

Supplementary Table 7. List of common founder mutations elicitating automatic germline genetic confirmation upon their detection in a tumor

Gene	Genetic variant with founder effect		Population	Reference	TGV found in our cohort (n of cases)[a]	Genetic Testing
	HGVS nomenclature	Alternate Names				
APC	NM_000038.6:c.3920T>A p.(Ile1307Lys)	I1307K	Ashkenazi Jewish	[14-16]	No	-
ATM	NM_000051.4:c.7271T>G p.(Val2424Gly)	V2424G	European	[1, 3, 18]	No	-
BRCA1	NM_007294.4:c.68_69del p.(Glu23ValfsTer17)	187delAG; 185delAG; 185_186delAG	Ashkenazi Jewish	[1, 14-16, 18]	No	-
	NM_007294.4:c.5266dup p.(Gln1756ProfsTer74)	5382_5383insC; 5382insC; 5384insC; 5385insC; 5383insC	Ashkenazi Jewish		No	-
BRCA2	NM_000059.4:c.5946del p.(Ser1982ArgfsTer22)	S1982fs; 6174delT	Ashkenazi Jewish	[1, 14-16, 18]	No	-
CHEK2	NM_007194.4:c.1100del p.(Thr367MetfsTer15)	T146fs; T338fs; T410fs; T300fs; T367fs	European	[3, 14-16, 18, 20]	No	-
	NC_000022.11(NM_007194.4):c.444+1G>A p.?	IVS3+1G>A; IVS2+1G>A	European		No	-
	NM_007194.4:c.470T>C p.(Ile157Thr)	I157T; I200T	European		Yes (n = 1)	1 True Germline
	NM_007194.4:c.1283C>T p.(Ser428Phe)	S428F; S399F; S207F; S361F; S471F	Ashkenazi Jewish		No	-
HOXB13	NM_006361.6:c.251G>A p.(Gly84Glu)	G84E	European	[17, 19]	Yes (n = 2)	2 Not Performed
MSH2	NM_000251.3:c.1906G>C p.(Ala636Pro)	A636P; A570P	Ashkenazi Jewish	[14, 16]	No	-
MSH6	NM_000179.3:c.3984_3987dup p.(Leu1330ValfsTer12)	3984_3987dupGTCA; L1200fs; L1028fs; L1330fs	Ashkenazi Jewish	[14, 16]	No	-
	NM_000179.3:c.3959_3962del p.(Ala1320GlufsTer6)	3959_3962delCAAG; A1190fs; A1018fs	Ashkenazi Jewish		No	-
MUTYH	NM_001048174.2:c.452A>G p.(Tyr151Cys)	536A>G; Y165C; Y179C; Y176C; Y151C; Y162C; Y152C; Y166C	European	[15, 18]	Yes (n = 2)[b]	2 Not Recommended
	NM_001048174.2:c.1103G>A p.(Gly368Asp)	1187G>A; G382D; G396D; G276D; G393D; G369D; G368D	European		Yes (n = 2)[b]	1 Not Performed<br>1 Not Recommended
TP53	NM_000546.6:c.1010G>A p.(Arg337His)	R337H; R205H; R298H; R178H	Brazilian	[3]	No	-

HGVS, Human Genome Variation Society; TGV, tumor genetic variants; True Germline, genetic testing was recommended, performed and germline origin was confirmed; Not Performed, genetic testing was recommended but not performed; Not Recommended, genetic testing was not recommended.

a: Number in parenthesis corresponds to unique cases. Total cases in our cohort with founder mutations found in their tumors were 7/83.

b: All TGVs in the gene *MUTYH* were observed without any other variant in the same gene. Two of the most known founder mutations in *MUTYH* (2 G368D and 2 Y151C) were reported in four cases.

Supplementary Table 8. Evaluation of 127 TGVs by the gMTB from 83 cases from OCTANE clinical trial, to determine which variants may be considered as *germline relevant*

Tumor genetic variant (TGV) characteristics	Total TGVs (n = 127)	Germline Relevant		p-value
		No (n = 83)	Yes (n = 44)	
**Variant Class, n (%)[a]**				0.305
Missense	39	22 (56.4%)	17 (43.6%)	
Nonsense (LOF)	34	22 (64.7%)	12 (35.3%)	
Frameshift (LOF)	31	22 (71.0%)	9 (29.0%)	
Intronic	17	11 (64.7%)	6 (35.3%)	
Other	6	6 (100%)	0	
**Tier Category in the somatic setting, n (%)[b]**				**0.003**
I	19	6 (31.6%)	13 (68.4%)	
II	81	56 (69.1%)	25 (30.9%)	
III	26	20 (76.9%)	6 (23.1%)	
**Clinical Actionability by ESMO, n (%)[a]**				**<0.001**
Most-actionable (MA)	16	1 (6.3%)	15 (93.8%)	
High-actionability (HA)	62	53 (85.5%)	9 (14.5%)	
Standard-actionability (SA)	37	22 (59.5%)	15 (40.5%)	
Not defined in ESMO[c]	12	7 (58.3%)	5 (41.7%)	
**Pathogenicity in ClinVar in the germline setting, n (%)[a]**				**0.045**
Pathogenic/likely pathogenic (P/LP)	78	45 (57.7%)	33 (42.3%)	
Variant of uncertain significance (VUS)	11	10 (90.9%)	1 (9.1%)[f]	
Conflicting/Not reported	38	28 (73.7%)	10 (26.3%)	
**Tumor Context, n (%) [a]**				0.121
On-tumor	65	38 (58.5%)	27 (41.5%)	

Off-tumor	50	38 (76.0%)	12 (24.0%)	
Not defined in ESMO[c]	12	7 (58.3%)	5 (41.7%)	
**Germline Conversion Rate (GCR), median% [Q1-Q3; n][d]**				**<0.001**
On-tumor	65	0.7% [0.7-3.6; n = 38]	22.0% [6.7-39.1; n = 27]	
Off-tumor	50	4.5% [1.2-45.4; n = 38]	78.7% [38.5-88.7; n = 12]	
Not defined in ESMO[c]	12	NA [n = 7]	NA [n = 5]	
**Variant Allele Fraction (VAF), median% [Q1-Q3; n][e]**				**0.021**
GGT not recommended	83	34.0% [25.5-51.5; n = 83]	NA	
GGT recommended - True somatic	18	NA	38.0% [30.8-51.8; n = 18]	
GGT recommended - Unknown	17	NA	40.0% [26.0-50.0; n = 17]	
GGT recommended - True germline	9	NA	59.0% [48.0-76.0; n = 9]	

TGV, tumor genetic variants; gMTB, germline molecular tumor board; OCTANE, the Ontario-wide Cancer Targeted Nucleic Acid Evaluation (OCTANE) clinical trial [11]; ESMO, The European Society of Medical Oncology (ESMO) Precision Medicine Working Group Germline Subgroup [1, 4]; LOF, loss-of-function genetic variants; GGT, germline genetic testing/confirmation; Q1, first quartile; Q3, third quartile; NA, not applicable.

a: Fisher-Freeman Halton test was used to investigate for differences in variant class, clinical actionability, pathogenicity in ClinVar, and tumor context.

b: χ^2 - test was used to assess for differences in Tier categories (I, II, and III). One *CHEK2* TGV [NM_007194.4:c.1556G>T p.(Arg519Leu)] was excluded from this comparison since the UHN laboratory did not determine any somatic interpretation.

c: Corresponds to 12 TGVs identified in cancer susceptibility genes not included in ESMO. For these, we did not assign any clinical actionability, tumor context or GCR. For p-value calculation of GCR by tumor context, these 12 were excluded.

d: Kruskal-Wallis rank sum test was used to assess differences in GCR (Shapiro-Wilk test p-value < 0.05; Levene's test p-value < 0.05). Pairwise comparisons using Dunn's test with p-values adjusted with the Holm method are shown in a separate file (Supplementary Material 2 - Post Hoc test 2).

e: Kruskal-Wallis rank sum test was used to assess differences in VAF (Shapiro-Wilk test p-value < 0.05 for the category 'GGT not recommended'; Levene's test p-value > 0.05). Pairwise comparisons using Dunn's test with p-values adjusted with the Holm method are shown in a separate file (Supplementary Material 2 - Post Hoc test 3). The median VAF of TGV in the *germline relevant* group was 46.0% [33.0-53.0; n = 44].

f: One VUS in *SDHAF2* was considered relevant on the germline NM_017841.4:c.444_447del p.(Asn148LysfsTer34). It has two submissions in ClinVar as VUS. Since it is a frameshift and in automated curation tools the interpretations were conflicting (VUS in Varsome and likely pathogenic in Franklin), it was recommended for germline confirmation.

Supplementary Table 9. Reasons that influenced compliance to genetic testing indicated for OCTANE cases assigned to Group 2 (a, b and c)

**Reasons**	**GGT not completed - Group 2 (n = 56)**			**Total Cases**	**Total Cases; TGV**	
	**Group 2a - Only met 'germline criteria'**	**Group 2b - Only met 'tumor-only criteria'**	**Group 2c - Met both criteria 'germline and tumor-only'**	**All Group 2 (n = 56)**	**Only Groups 2b and 2c**	
	**Cases (5/20); TGV (NA)**	**Cases (n = 7/9); TGV (n = 8)**	**Cases (n = 7/27) TGV (n = 9)**		**Cases**	**TGV**
Patient died	1 case[a]	4 cases (5 TGV)	2 cases (3 TGV)	7	6	8
GGT not ordered by MD	3 cases	1 case (1 TGV)	1 case (1 TGV)[b]	5	2	2
GGT ordered & pending	1 case	2 cases (2 TGV)	3 cases (3 TGV)[b]	6	5	5
GGT was rejected by the patient		-	1 case (2 TGV)	1	1	2
**Total**				**19[c]**	**14**	**17**

OCTANE, the Ontario-wide Cancer Targeted Nucleic Acid Evaluation (OCTANE) clinical trial [11]; GGT, germline genetic testing; TGV, tumor genetic variants; Germline criteria, based on the Cancer Care Ontario Hereditary Cancer Testing Eligibility Criteria [14]; Tumor-only criteria, the TGV met the recommendation for germline confirmation as per tumor-only guidelines [1-10]; NA, Not applicable; MD, medical oncologist or responsible clinician

a: This case was also out of Ontario Province

b: One case in each of these categories did not have any GGT performed at any point (before or after tumor testing and comprehensive multi-disciplinary case review by the germline Molecular Tumor Board (gMTB))

c: Overall, 14/19 did not have any GGT done before or after tumor testing and case review by the gMTB (5 cases in Group 2a; 7 cases in Group 2b and 2 cases in Group 2c[b]). The other 5/19 cases who had a previous genetic testing result, were included in this table because additional recommendations for specific mutations or an extended panel were not completed.

REFERENCES

1. Mandelker D, Donoghue M, Talukdar S, Bandlamudi C, Srinivasan P, Vivek M, et al. Germline-focussed analysis of tumour-only sequencing: recommendations from the ESMO Precision Medicine Working Group. Ann Oncol. 2019; 30(8):1221-1231. doi:10.1093/annonc/mdz136
2. Lincoln SE, Nussbaum RL, Kurian AW, Nielsen SM, Das K, Michalski S, et al. Yield and Utility of Germline Testing Following Tumor Sequencing in Patients With Cancer. JAMA Netw Open. 2020; 3(10):e2019452. doi:10.1001/jamanetworkopen.2020.19452
3. Clark DF, Maxwell KN, Powers J, Lieberman DB, Ebrahimzadeh J, Long JM, et al. Identification and Confirmation of Potentially Actionable Germline Mutations in Tumor-Only Genomic Sequencing. JCO Precis Oncol. 2019; 3. doi:10.1200/PO.19.00076
4. Kuzbari Z, Bandlamudi C, Loveday C, Garrett A, Mehine M, George A, et al. Germline-focused analysis of tumour-detected variants in 49,264 cancer patients: ESMO Precision Medicine Working Group recommendations. Ann Oncol. 2023; 34(3):215-227. doi:10.1016/j.annonc.2022.12.003
5. Directors ABo. ACMG policy statement: updated recommendations regarding analysis and reporting of secondary findings in clinical genome-scale sequencing. Genet Med. 2015; 17(1):68-69. doi:10.1038/gim.2014.151
6. Cushman-Vokoun A, Lauring J, Pfeifer J, Olson DR, Berry A, Thorson J, et al. Laboratory and Clinical Implications of Incidental and Secondary Germline Findings During Tumor Testing. Arch Pathol Lab Med. 2022; 146(1):70-77. doi:10.5858/arpa.2020-0025-CP
7. Jalloul N, Gomy I, Stokes S, Gusev A, Johnson BE, Lindeman NI, et al. Germline Testing Data Validate Inferences of Mutational Status for Variants Detected From Tumor-Only Sequencing. JCO Precis Oncol. 2021; 5. doi:10.1200/PO.21.00279
8. Schienda J, Church AJ, Corson LB, Decker B, Clinton CM, Manning DK, et al. Germline Sequencing Improves Tumor-Only Sequencing Interpretation in a Precision Genomic Study of Patients With Pediatric Solid Tumor. JCO Precis Oncol. 2021; 5. doi:10.1200/PO.21.00281
9. Klek S, Heald B, Milinovich A, Ni Y, Abraham J, Mahdi H, et al. Genetic Counseling and Germline Testing in the Era of Tumor Sequencing: A Cohort Study. JNCI Cancer Spectr. 2020; 4(3):pkaa018. doi:10.1093/jncics/pkaa018
10. Mutetwa T, Goudie C, Foulkes WD, Polak P. Companion Tumor Sequencing to Assess the Clinical Significance of Germline Sequencing in Children With Cancer. JAMA Netw Open. 2021; 4(11):e2135135. doi:10.1001/jamanetworkopen.2021.35135
11. Malone ER, Saleh RR, Yu C, Ahmed L, Pugh T, Torchia J, et al. OCTANE (Ontario-wide Cancer Targeted Nucleic Acid Evaluation): a platform for intraprovincial, national, and international clinical data-sharing. Curr Oncol. 2019; 26(5):e618-e623. doi:10.3747/co.26.5235
12. Montgomery ND, Selitsky SR, Patel NM, Hayes DN, Parker JS, Weck KE. Identification of Germline Variants in Tumor Genomic Sequencing Analysis. J Mol Diagn. 2018; 20(1):123-125. doi:10.1016/j.jmoldx.2017.09.008
13. Boscolo Bielo L, Trapani D, Repetto M, Crimini E, Valenza C, Belli C, et al. Variant allele frequency: a decision-making tool in precision oncology? Trends Cancer. 2023; 9(12):1058-1068. doi:10.1016/j.trecan.2023.08.011
14. Bell KA, Kim R, Aronson M, Gillies B, Ali Awan A, Chun K, et al. Development of a comprehensive approach to adult hereditary cancer testing in Ontario. J Med Genet. 2023; 60(8):769-775. doi:10.1136/jmg-2022-108945
15. DeLeonardis K, Hogan L, Cannistra SA, Rangachari D, Tung N. When Should Tumor Genomic Profiling Prompt Consideration of Germline Testing? J Oncol Pract. 2019; 15(9):465-473. doi:10.1200/JOP.19.00201
16. Mandelker D, Zhang L, Kemel Y, Stadler ZK, Joseph V, Zehir A, et al. Mutation Detection in Patients With Advanced Cancer by Universal Sequencing of Cancer-Related Genes in Tumor and Normal DNA vs Guideline-Based Germline Testing. JAMA. 2017; 318(9):825-835. doi:10.1001/jama.2017.11137
17. Truong H, Breen K, Nandakumar S, Sjoberg DD, Kemel Y, Mehta N, et al. Gene-based Confirmatory Germline Testing Following Tumor-only Sequencing of Prostate Cancer. Eur Urol. 2023; 83(1):29-38. doi:10.1016/j.eururo.2022.08.028
18. Tung N, Dougherty KC, Gatof ES, DeLeonardis K, Hogan L, Tukachinsky H, et al. Potential pathogenic germline variant reporting from tumor comprehensive genomic profiling complements classic approaches to germline testing. NPJ Precis Oncol. 2023; 7(1):76. doi:10.1038/s41698-023-00429-1
19. Xu J, Lange EM, Lu L, Zheng SL, Wang Z, Thibodeau SN, et al. HOXB13 is a susceptibility gene for prostate cancer: results from the International Consortium for Prostate Cancer Genetics (ICPCG). Hum Genet. 2013; 132(1):5-14. doi:10.1007/s00439-012-1229-4
20. Sutcliffe EG, Stettner AR, Miller SA, Solomon SR, Marshall ML, Roberts ME, et al. Differences in cancer prevalence among CHEK2 carriers identified via multi-gene panel testing. Cancer Genet. 2020; 246-247:12-17. doi:10.1016/j.cancergen.2020.07.001
