# Supplementary material for: Clinical integration of germline findings from a tumor testing precision medicine program
Source: BMC Cancer. 2025 Jan 30;25:176. doi: 10.1186/s12885-025-13487-4 (PMC11783960; doi:10.1186/s12885-025-13487-4)
Supplement: Supplementary file 2 — Supplementary Material 2 [file 12885_2025_13487_MOESM2_ESM.docx]

Supplementary Material 2

Post Hoc test 1. Pairwise comparisons using Tukey’s HSD Test after significant ANOVA p-value showing mean differences of age at diagnosis between groups

| **Tukey multiple comparison test** | **Mean differences^a^** | **95% Confidence Intervals^b^** | | **Adjusted p-values^c^** |
| --- | --- | --- | --- | --- |
|  |  | **Lower** | **Upper** |  |
| Group 2a - Group 1 | -2.50 | -13.46 | 8.46 | 0.932 |
| Group 2b - Group 1 | -6.04 | -20.33 | 8.26 | 0.686 |
| **Group 2c - Group 1** | **-13.44** | **-23.55** | **-3.34** | **0.004** |
| Group 2b - Group 2a | -3.54 | -18.45 | 11.37 | 0.924 |
| **Group 2c - Group 2a** | **-10.95** | **-21.90** | **0.01** | **0.050** |
| Group 2c - Group 2b | -7.41 | -21.70 | 6.89 | 0.528 |

Tukey's HSD test, Tukey's Honestly Significant Difference test; Group 1, GGT - Not recommended; Group 2a, GGT - Recommended (‘germline criteria’); (Group 2b), GGT - Recommended (‘tumor-only criteria’); Group 2c, GGT - Recommended (‘germline and tumor-only criteria’); GGT, germline genetic testing/confirmation; Germline criteria, patients met the Cancer Care Ontario Hereditary Cancer Testing Eligibility Criteria; TGV, tumor genetic variant; Tumor-only criteria, the TGV met the recommendation for germline confirmation as per tumor-only guidelines [1-10].

a: Estimated difference of the mean age at diagnosis between each pair of groups to determine which group is significantly different from the others (in relation to Table 1)

b: Corresponding 95% confidence intervals

c: Adjusted p-values to control the family-wise error rate at the 0.05 level across multiple pair-wise comparisons. Values ≤0.05 are in bold.

Post Hoc test 2. Pairwise comparisons using Dunn's test after significant Kruskal-Wallis p-value showing differences in median GCR between groups and tumor context

| **Dunn's multiple comparison test** | **Z^a^** | **Unadjusted p-values** | **Adjusted p-values^b^** |  |
| --- | --- | --- | --- | --- |
|  |  |  |  |  |
| **Not *germline relevant* [Off-tumor] - Not *germline relevant* [On-tumor]** | **3.54** | **0.000** | **0.002** |  |
| **Not *germline relevant* [Off-tumor] - *Germline relevant* [Off-tumor]** | **-2.90** | **0.004** | **0.011** |  |
| **Not *germline relevant* [On-tumor] - *Germline relevant* [Off-tumor]** | **-5.35** | **0.000** | **0.000** |  |
| Not *germline relevant* [Off-tumor] - *Germline relevant* [On-tumor] | -1.77 | 0.077 | 0.154 |  |
| **Not *germline relevant* [On-tumor] - *Germline relevant* [On-tumor]** | **-4.99** | **0.000** | **0.000** |  |
| *Germline relevant* [Off-tumor] - *Germline relevant* [On-tumor] | 1.49 | 0.136 | 0.136 |  |

GCR, germline conversion rate; Not *germline relevant,* corresponds to TGVs not recommended for GGT; *Germline relevant*, corresponds to TGVs recommended for GGT; TGV, tumor genetic variant; GGT, germline genetic testing/confirmation.

a: Values for the Z-test statistic for each comparison to determine which group is significantly different from the others (in relation to Supplementary Material 1 - Table 8)

b: Holm method was used to obtained adjusted p-values to control the family-wise error rate at the 0.05 level across multiple pair-wise comparisons. Values ≤0.05 are in bold

Post Hoc test 3. Pairwise comparisons using Dunn's test after significant Kruskal-Wallis p-value showing differences in median VAF between groups

| **Dunn's multiple comparison test** | **Z^a^** | **Unadjusted p-values** | **Adjusted p-values^b^** |  |
| --- | --- | --- | --- | --- |
|  |  |  |  |  |
| GGT True somatic - GGT Not recommended | 0.74 | 0.462 | 1.000 |  |
| GGT True somatic - GGT Unknown | 0.20 | 0.843 | 0.843 |  |
| GGT True somatic - GGT True germline | -2.20 | 0.028 | 0.111 |  |
| GGT Not recommended - GGT Unknown | -0.47 | 0.641 | 1.000 |  |
| **GGT Not recommended - GGT True germline** | **-3.11** | **0.002** | **0.011** |  |
| GGT Unknown - GGT True germline | -2.34 | 0.019 | 0.095 |  |

VAF, variant allele fraction, GGT, germline genetic testing/confirmation; GGT Not recommended, this category refers to TGV considered *not germline relevant*; TGV, tumor genetic variant; The following three categories (GGT True somatic, GGT True germline, and GGT Unknown) are all within the group of TGVs considered *germline relevant*.

a: Values for the Z-test statistic for each comparison to determine which group is significantly different from the others (in relation to Supplementary Material 1 - Table 8)

b: Holm method was used to obtained adjusted p-values to control the family-wise error rate at the 0.05 level across multiple pair-wise comparisons. Values ≤0.05 are in bold

REFERENCES

1. Directors ABo. ACMG policy statement: updated recommendations regarding analysis and reporting of secondary findings in clinical genome-scale sequencing. Genet Med. 2015; 17(1):68-69. doi:10.1038/gim.2014.151

2. Mandelker D, Donoghue M, Talukdar S, Bandlamudi C, Srinivasan P, Vivek M, et al. Germline-focussed analysis of tumour-only sequencing: recommendations from the ESMO Precision Medicine Working Group. Ann Oncol. 2019; 30(8):1221-1231. doi:10.1093/annonc/mdz136

3. Clark DF, Maxwell KN, Powers J, Lieberman DB, Ebrahimzadeh J, Long JM, et al. Identification and Confirmation of Potentially Actionable Germline Mutations in Tumor-Only Genomic Sequencing. JCO Precis Oncol. 2019; 3. doi:10.1200/PO.19.00076

4. Klek S, Heald B, Milinovich A, Ni Y, Abraham J, Mahdi H, et al. Genetic Counseling and Germline Testing in the Era of Tumor Sequencing: A Cohort Study. JNCI Cancer Spectr. 2020; 4(3):pkaa018. doi:10.1093/jncics/pkaa018

5. Lincoln SE, Nussbaum RL, Kurian AW, Nielsen SM, Das K, Michalski S, et al. Yield and Utility of Germline Testing Following Tumor Sequencing in Patients With Cancer. JAMA Netw Open. 2020; 3(10):e2019452. doi:10.1001/jamanetworkopen.2020.19452

6. Cushman-Vokoun A, Lauring J, Pfeifer J, Olson DR, Berry A, Thorson J, et al. Laboratory and Clinical Implications of Incidental and Secondary Germline Findings During Tumor Testing. Arch Pathol Lab Med. 2022; 146(1):70-77. doi:10.5858/arpa.2020-0025-CP

7. Mutetwa T, Goudie C, Foulkes WD, Polak P. Companion Tumor Sequencing to Assess the Clinical Significance of Germline Sequencing in Children With Cancer. JAMA Netw Open. 2021; 4(11):e2135135. doi:10.1001/jamanetworkopen.2021.35135

8. Jalloul N, Gomy I, Stokes S, Gusev A, Johnson BE, Lindeman NI, et al. Germline Testing Data Validate Inferences of Mutational Status for Variants Detected From Tumor-Only Sequencing. JCO Precis Oncol. 2021; 5. doi:10.1200/PO.21.00279

9. Schienda J, Church AJ, Corson LB, Decker B, Clinton CM, Manning DK, et al. Germline Sequencing Improves Tumor-Only Sequencing Interpretation in a Precision Genomic Study of Patients With Pediatric Solid Tumor. JCO Precis Oncol. 2021; 5. doi:10.1200/PO.21.00281

10. Kuzbari Z, Bandlamudi C, Loveday C, Garrett A, Mehine M, George A, et al. Germline-focused analysis of tumour-detected variants in 49,264 cancer patients: ESMO Precision Medicine Working Group recommendations. Ann Oncol. 2023; 34(3):215-227. doi:10.1016/j.annonc.2022.12.003
